# Clinical Significance of *TP53*-Mutant Clonal Hematopoiesis Across Diseases

**DOI:** 10.1158/2643-3230.BCD-24-0355

**Published:** 2025-06-17

**Authors:** Yoshiaki Usui, Mikiko Endo, Yusuke Iwasaki, Hanae Iijima, Hidewaki Nakagawa, Koichi Matsuda, Yukihide Momozawa

**Affiliations:** 1 Laboratory for Genotyping Development, RIKEN Center for Integrative Medical Sciences, Yokohama, Japan.; 2 Laboratory for Cancer Genomics, RIKEN Center for Integrative Medical Sciences, Yokohama, Japan.; 3 Laboratory of Clinical Genome Sequencing, Department of Computational Biology and Medical Sciences, Graduate School of Frontier Sciences, The University of Tokyo, Tokyo, Japan.

## Abstract

Clonal hematopoiesis of indeterminate potential (CHIP) has broad clinical relevance, and *TP53* plays various roles within cells. However, the gene-specific and cross-disease significance of CHIP with *TP53* mutations (*TP53*-CHIP) remains unclear. In this study, we evaluated *TP53*-CHIP using targeted sequencing data of 140,597 individuals without hematologic neoplasms in BioBank Japan. We identified 1,157 individuals with *TP53*-CHIP and clarified the characteristics of mutations and carriers. *TP53*-CHIP was associated with poor overall survival, especially because of lymphoid neoplasms and respiratory disease, in addition to myeloid neoplasms. Significant interactions accompanied by excess risks were observed between *TP53*-CHIP and lifestyle factors for disease-specific mortality: acetaldehyde exposure (resulting from the interaction between drinking and the germline variant of *ALDH2*) for myeloid neoplasms and smoking for respiratory disease. The clinical significance of *TP53*-CHIP was sometimes largely independent of variant allele fractions. These findings elucidate aspects of disease pathogenesis and inform personalized risk management.

**Significance:**

*TP53*-CHIP contributed to a wide range of outcomes besides myeloid neoplasm mortality. *TP53*-CHIP, when combined with environmental factors, showed a remarkably higher risk for disease-specific mortality, accompanied by excess risks.

## Teaser

A large-scale population study links *TP53*-mutant clonal hematopoiesis to increased risk of hematologic malignancies and respiratory mortality, with contributions of germline variants, environmental factors, and somatic mutations.

## Introduction

Clonal hematopoiesis of indeterminate potential (CHIP) is commonly defined as clonally expanded somatic mutations of variant allele fractions (VAF) to 2% or more in multiple blood lineages or in hematopoietic stem cells in the absence of hematologic neoplasms (1). It has a wide clinical significance, including an association with the risk of myeloid neoplasms (1). Furthermore, it is frequently encountered in the clinical sequencing for individuals with cancer (2). The molecular and clinical consequences of somatic mutations differ depending on the genes affected, suggesting that a gene-specific approach is often more informative than broad categorizations (3). Evaluating CHIP at the gene level alongside clinical data has the potential to enhance risk assessment and clarify disease mechanisms. Moreover, integrating evidence across diseases is crucial for providing comprehensive carrier management.

The *TP53* gene encodes the p53 tumor suppressor protein, which is commonly referred to as the “Guardian of the Genome” (4). *TP53* plays various roles within cells, and its mutation causes various alterations in hematopoietic stem and progenitor cell function (1). Although *TP53* is a biologically important gene and is often affected in CHIP, its clinical relevance beyond myeloid neoplasms (1, 5) and cancer treatment (1, 2, 6) is unclear due to an inadequate evaluation. One reason for this is that the prevalence of CHIP with *TP53* mutations (*TP53*-CHIP) is low (0.11% according to the UK BioBank report; ref. 7). For comparison, the prevalence of CHIP with mutations in *DNMT3A*, the most frequently mutated gene in CHIP, was 4.11% (7). Another reason is that conventional sequence data have a detection sensitivity below half of what is expected for mutations with VAFs less than 5% (8), leading to underestimation. Indeed, the previous studies included only a limited number of *TP53*-CHIP carriers (up to approximately 500 carriers in the same cohort; refs. 5, 7, 8), resulting in insufficient data on *TP53*-CHIP.

We hypothesized that *TP53*-CHIP contributes to a wide range of clinical outcomes. To explore this, we conducted a large-scale and high-resolution evaluation of *TP53*-CHIP across diseases, providing novel insights into disease pathogenesis and clinical management.

## Results

### Cohort Description

The characteristics of 140,597 study participants are shown in Supplementary Table S1. The mean age was 65.48 years. The proportion of male participants was 55.77%. The proportions of comorbidities were 51.43% for cancer, 26.49% for hypertension, 29.15% for hyperlipidemia, and 24.60% for diabetes. Although the proportion of individuals with cancer was high, the study cohort did not seem to deviate markedly from the general characteristics of the overall BioBank Japan cohort (9–11). A total of 81,462 individuals were included in the follow-up survival survey (median follow-up time: 9.95 years).

### Detection of *TP53*-CHIP

**Figure 1.**
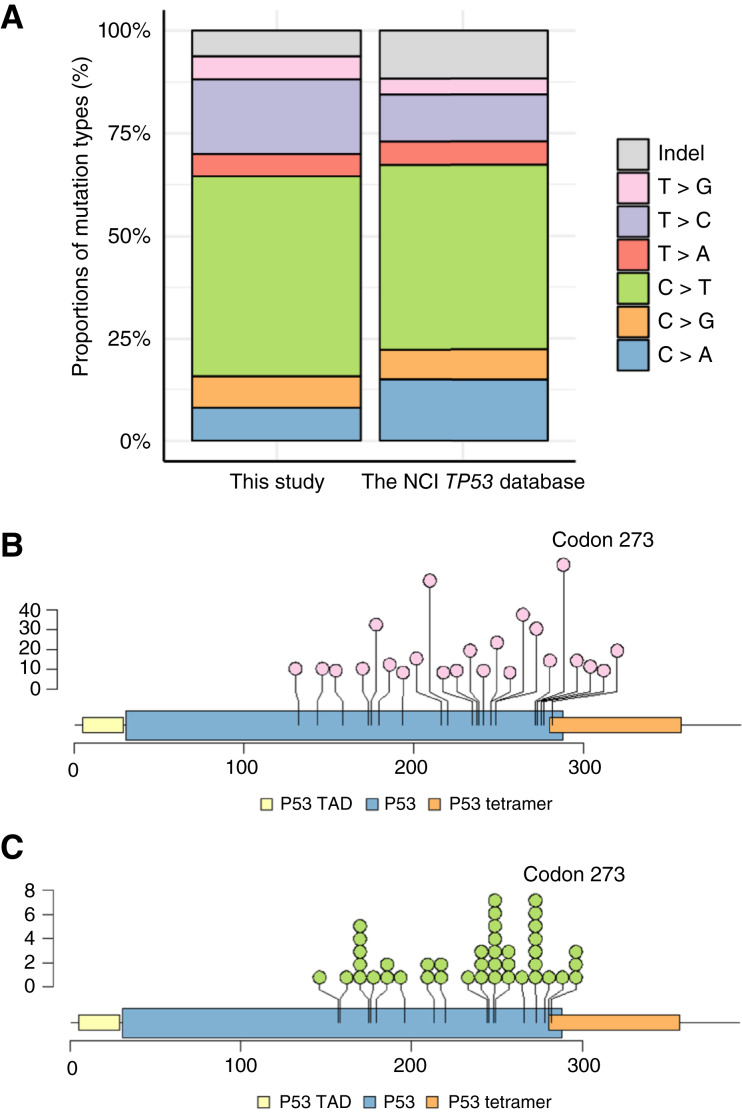
Mutation patterns and hotspot locations of *TP53*-CHIP. **A,** Mutation patterns of CHIP in this study (1,198 mutations) and somatic mutations in the NCI *TP53* database (RRID: SCR_026878; 28,310 mutations). **B,** Mutations that 10 or more individuals with *TP53*-CHIP carry in this study are shown. The vertical axis represents the number of carriers. Detailed information of mutations is provided in Supplementary Table S2. **C,** Codon of the hotspot mutation, which accounted for more than 1% of all mutations, in the NCI *TP53* database (RRID: SCR_026878). The vertical axis represents the proportion of mutations at each codon. Indel, insertion or deletion.

The average coverage was 1,621 sequence reads. More than 99% of the targeted region (1,311 bp) was covered by 100 sequence reads in more than 95% of the participants. We identified 421 distinct mutations among 1,157 individuals in this study (Supplementary Table S2). None of these were present among variants with minor allele frequencies 0.5% or more in Genome Aggregation Database (gnomAD; RRID: SCR_014964). Through droplet digital PCR (ddPCR) analyses for detected hotspot mutations, we observed little fixed and proportional bias (Supplementary Fig. S1), and there was a very high intraclass correlation coefficient between the targeted sequencing and ddPCR [0.992; 95% confidence interval (CI), 0.991–0.994]. The mutation patterns in this study, the locations of hotspot mutations, and somatic mutations in the NCI database (RRID: SCR_026878) for comparison are shown in Fig. 1. All hotspot mutations in 10 or more individuals with CHIP were missense mutations (Supplementary Table S2). Approximately half of the mutations identified in CHIP were C>T, which is similar to the somatic pattern reported in the NCI database (RRID: SCR_026878; Fig. 1A). In both, the most frequently mutated location in *TP53* was codon 273 (Fig. 1B and C).

### Characteristics of *TP53*-CHIP Carriers and Noncarriers

The prevalence of *TP53*-CHIP with VAFs of 2% or more remarkably increased with age [0.09%, 0.12%, 0.26%, 0.62%, 1.19%, and 1.94% among individuals <40, 50–59, 60–69, 70–79, and ≥80 years of age, respectively; *P* for trend (Cochran–Armitage test) = 2.25 × 10^−12^]. This trend was more pronounced even within the low VAF subgroup (2% to <5%; Supplementary Fig. S2A). The prevalence was higher in males than in females [*P* (*χ*^2^ test) = 1.25 × 10^−21^], and the increasing trend with age was more pronounced in males than in females (Supplementary Fig. S2B). Although the prevalence was higher among those with cancer [*P* (*χ*^2^ test) = 2.56 × 10^−11^], the prevalence among those without cancer also remarkably increased with age (Supplementary Fig. S2C).

We focused on environmental risk factors for *TP53*-CHIP (Supplementary Table S3). The germline variant *ALDH2* rs671, an East Asian–specific common variant known for its role in ethanol metabolism (12), was also sequenced using a multiplex PCR-based targeted sequencing method (13). Smoking habits were associated with *TP53*-CHIP [OR, 1.37; 95% CI, 1.18–1.59; *P* (Wald test) = 3.43 × 10^−5^] and depended on the smoking dose [*P* (Wald test) = 2.95 × 10^−5^]. No significant association was found between drinking habits and *TP53*-CHIP in the overall cohort [OR, 1.11; 95% CI, 0.97–1.27; *P* (Wald test) = 0.128]. However, drinking habits and *ALDH2* rs671 showed a significant gene–environment interaction with regard to the presence of *TP53*-CHIP [*P* for interaction (Wald test) = 1.10 × 10^−4^]. In individuals with *ALDH2* rs671 Lys+, who exhibit a lower enzyme activity for detoxifying acetaldehyde (12), drinking habits were significantly associated with *TP53*-CHIP [OR, 1.43; 95% CI, 1.19–1.73; *P* (Wald test) = 1.75 × 10^−4^], and this association was more pronounced with alcohol consumption dose [*P* (Wald test) = 3.11 × 10^−7^]. Among individuals with rs671 Lys+, the proportion of T>C observed in *TP53* was higher with alcohol consumption, although the difference was not statistically significant (Supplementary Fig. S3).

We did not observe a difference in the proportions of abnormal blood cell counts between *TP53*-CHIP noncarriers and carriers [15.29% (10,291/67,313) vs. 13.02% (75/576), *P* (*χ*^2^ test) = 0.132]. In addition, no association was observed between the blood cell count and VAFs among *TP53*-CHIP carriers [white blood cells: *P* (Wald test) = 0.091; hemoglobin: *P* (Wald test) = 0.425; hematocrit: *P* (Wald test) = 0.372; and platelets: *P* (Wald test) = 0.528].

### Clinical Significance of *TP53*-CHIP

**Figure 2.**
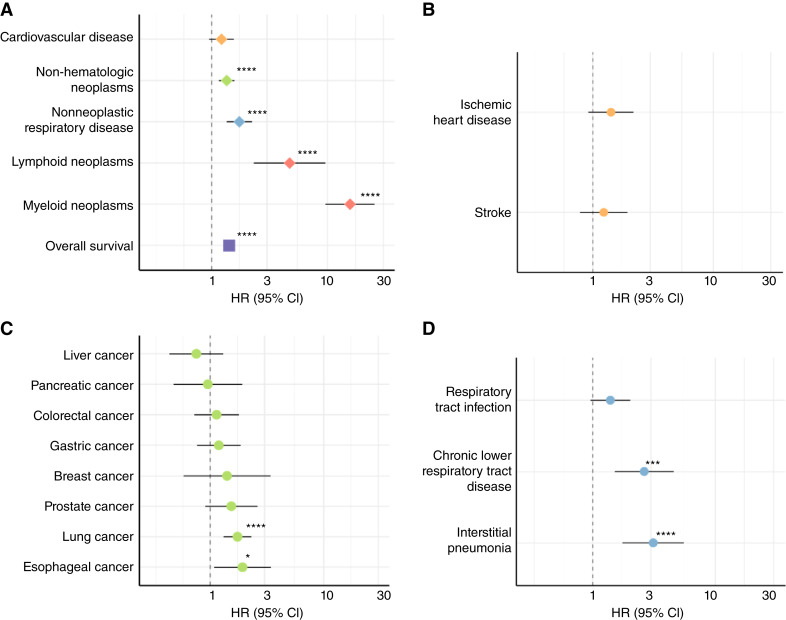
Clinical impact of *TP53*-CHIP. **A**, *TP53*-CHIP risk of mortality overall and across major disease categories (number of events: cardiovascular disease, 5,645; non-hematologic neoplasms, 13,256; nonneoplastic respiratory disease, 3,405; lymphoid neoplasms, 199; myeloid neoplasms, 162; and overall, 29,626). **B,** Sub-analysis of cardiovascular diseases (number of events: ischemic heart disease, 1,630; stroke, 1,649). **C,** Sub-analysis of non-hematologic neoplasms (number of events: liver cancer, 1,718; pancreatic cancer, 929; colorectal cancer, 1,897; gastric cancer, 1,751; breast cancer, 832; prostate cancer, 770; lung cancer, 2,833; and esophageal cancer, 593). **D,** Sub-analysis of nonneoplastic respiratory diseases (number of events: respiratory tract infection, 1,892; chronic lower respiratory tract disease, 440; and interstitial pneumonia, 410) are shown. HRs and their 95% CIs were estimated using Cox proportional hazards models of disease-specific mortality adjusted for age, sex, drinking habits, alcohol consumption, smoking habits, Brinkman index, body mass index, and comorbidities (hyperlipidemia, hypertension, diabetes, and cancer). All results among other models are shown in Supplementary Table S5. *, *q* < 0.1; ***, *q* < 0.01, and ****, *q* < 0.001 (Benjamini–Hochberg multiple test correction).

We performed a survival analysis to evaluate the clinical impact of *TP53*-CHIP. *TP53*-CHIP was associated with poor overall survival [HR, 1.42; 95% CI, 1.28–1.57; *P* (Wald test) = 9.85 × 10^−12^; Supplementary Fig. S4A; Fig. 2A]. We listed the causes of death for evaluating disease-specific mortality based on the predetermined criteria (Supplementary Table S4). The probabilities of the cumulative incidence of disease-specific mortality through 10 years are shown in Supplementary Fig. S4B and S4C. The incidence of mortality from hematologic neoplasms was markedly higher in the *TP53*-CHIP carriers than in the noncarriers [myeloid neoplasms: 2.78% vs. 0.15%, *P* (Gray test) = 8.75 × 10^−29^; lymphoid neoplasms: 1.30% vs. 0.21%, *P* (Gray test) = 4.22 × 10^−6^]. Multivariable analyses revealed that *TP53*-CHIP exerted different effects across diseases (Fig. 2A; Supplementary Table S5). *TP53*-CHIP was associated with non-hematologic neoplasms [Fig. 2A; HR, 1.35; 95% CI, 1.16–1.58; *P* (Wald test) = 1.50 × 10^−4^] and nonneoplastic respiratory disease mortality [Fig. 2A; HR, 1.73; 95% CI, 1.35–2.23; *P* (Wald test) = 1.85 × 10^−4^], in addition to myeloid neoplasms [Fig. 2A; HR, 15.54; 95% CI, 9.56–25.28; *P* (Wald test) = 2.83 × 10^−26^] and lymphoid neoplasms [Fig. 2A; HR, 4.70; 95% CI, 2.31–9.56; *P* (Wald test) = 1.98 × 10^−5^]. The association for myeloid neoplasm mortality was also observed in the analysis involving only individuals without cancer [HR, 8.00; 95% CI, 3.20–19.98; *P* (Wald test) = 8.44 × 10^−6^], indicating that the association was not solely attributable to secondary myeloid neoplasms. Although *TP53*-CHIP has been associated with an increased risk of hypodiploid acute lymphoblastic leukemia (14), no *TP53*-CHIP carriers were detected in this study population, in which several individuals died of acute lymphoblastic leukemia [International Classification of Diseases, 10th Revision (ICD-10), C91.0]. There was no significant association between *TP53*-CHIP and cardiovascular disease mortality [Fig. 2A; HR, 1.22; 95% CI, 0.95–1.56; *P* (Wald test) = 0.117] or with specific types of cardiovascular disease (Fig. 2B). Sub-analysis revealed that *TP53*-CHIP was associated with lung cancer mortality [Fig. 2C; HR, 1.74; 95% CI, 1.31–2.30; *P* (Wald test) = 1.12 × 10^−4^], chronic lower respiratory tract disease mortality [Fig. 2D; HR, 2.64; 95% CI, 1.52–4.59; *P* (Wald test) = 5.93 × 10^−4^], and interstitial pneumonia mortality [Fig. 2D; HR, 3.12; 95% CI, 1.76–5.56; *P* (Wald test) = 1.07 × 10^−4^]. Furthermore, it exhibited a trend of association with esophageal cancer mortality [Fig. 2C; HR, 1.92; 95% CI, 1.08–3.41; *P* (Wald test) = 0.025]. Sensitivity analysis considering the *ALDH2* genotype showed almost the same point estimate for all outcomes, except for esophageal cancer mortality (Supplementary Fig. S5). These associations were also observed when carriers with mutations registered in ClinVar (RRID: SCR_006169) as pathogenic/likely pathogenic and VAFs of 25% or more were excluded (Supplementary Fig. S6A and S6B).

**Figure 3.**
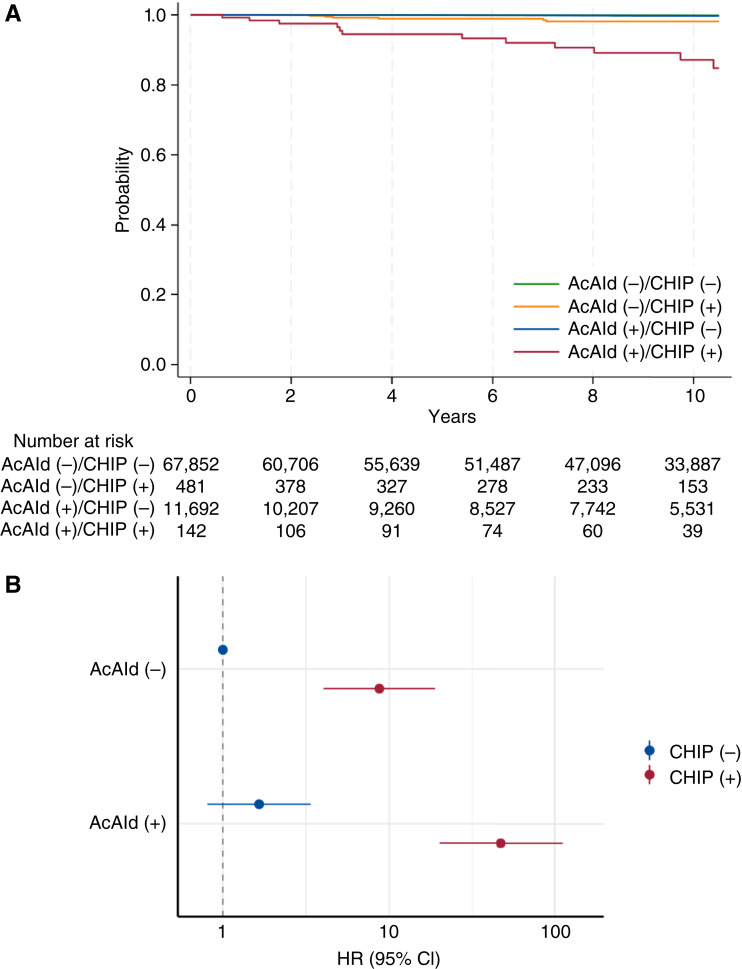
Impact of acetaldehyde and *TP53*-CHIP on myeloid neoplasm mortality. **A,** The cause-specific survival from myeloid neoplasms was estimated using the Kaplan–Meier method. Individuals prone to acetaldehyde exposure were defined as individuals with both rs671 Lys+ and ever-drinker status. **B,** HRs and their 95% CIs were estimated using Cox proportional hazards models of myeloid neoplasm mortality adjusted for age, sex, drinking habits, alcohol consumption, smoking habits, Brinkman index, body mass index, comorbidities (hyperlipidemia, hypertension, diabetes, and cancer), and *ALDH2*. AcAld, acetaldehyde.

**Figure 4.**
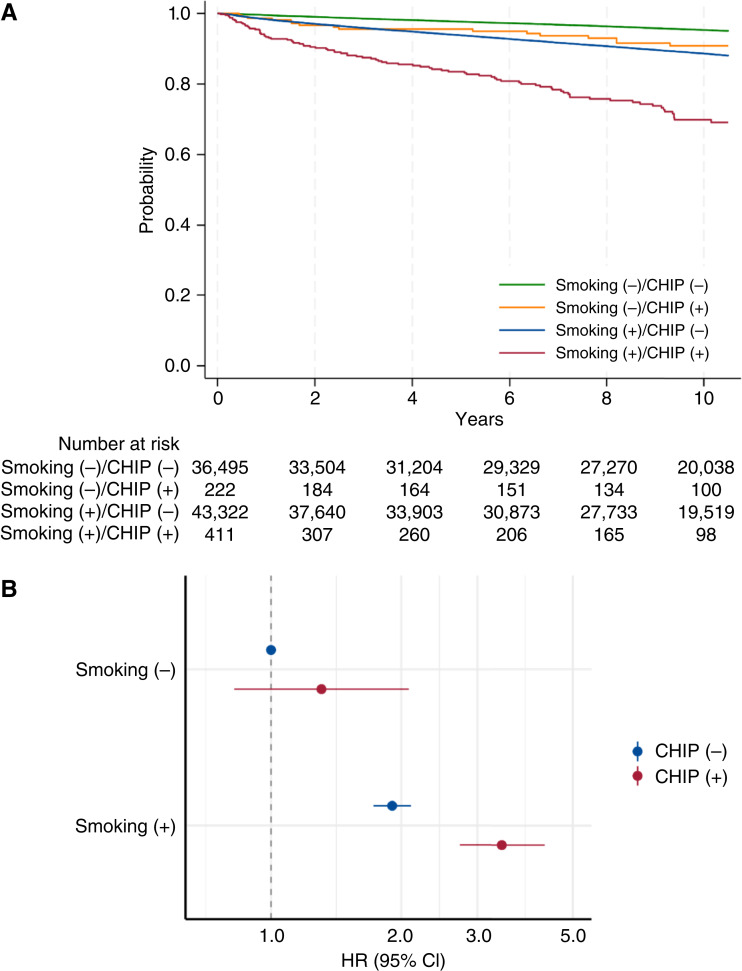
Impact of smoking and *TP53*-CHIP on respiratory disease mortality. **A,** The cause-specific survival from respiratory disease (nonneoplastic respiratory disease and lung cancer) was estimated using the Kaplan–Meier method. Smoking status was defined as ever smoker. **B,** HRs and their 95% CIs were estimated using Cox proportional hazards models of respiratory disease mortality adjusted for age, sex, drinking habits, alcohol consumption, Brinkman index, body mass index, and comorbidities (hyperlipidemia, hypertension, diabetes, and cancer).

Stratified analysis by environmental factors (acetaldehyde exposure or smoking) revealed their significant interactions with *TP53*-CHIP for myeloid neoplasms and respiratory disease (nonneoplastic respiratory disease and lung cancer) mortality (Supplementary Table S6). Individuals with acetaldehyde exposure and *TP53*-CHIP showed a remarkably higher risk of myeloid neoplasm mortality (HR, 47.37; 95% CI, 20.09–111.69) than those with *TP53*-CHIP alone (HR, 8.74; 95% CI, 4.05–18.88; Fig. 3A and B). Also, although smoking habits without *TP53*-CHIP carried a risk of respiratory disease mortality (HR, 1.91; (95% CI, 1.72–2.11), the risk was higher in the presence of *TP53*-CHIP (HR, 3.43; 95% CI, 2.73–4.30; Fig. 4A and B). When considering a genetic proxy for IL-6 inhibition (15), among individuals with smoking habits, individuals with the germline variant *IL6R* rs2228145 Ala+ tended to be less affected by *TP53*-CHIP (Supplementary Fig. S7A and S7B). In mediation analyses, we also observed interactions between environmental factors and *TP53*-CHIP for disease-specific mortality that operated only if *TP53*-CHIP was present (Supplementary Fig. S8A and S8B). These interactions were not attributable to *TP53*-CHIP serving as an intermediate factor of environmental factors, supporting independent interactions of these environmental factors (Supplementary Fig. S8A and S8B). Moreover, *TP53*-CHIP did not play a significant role as a pure intermediate factor between environmental factors and disease-specific mortality (Supplementary Fig. S8A and S8B).

With regard to myeloid neoplasm mortality, HRs remarkably increased proportionally to VAFs [2% to <5%, 9.43 (95% CI, 4.15–21.43); 5% to <10%, 12.85 (95% CI, 4.72–34.96); 10% to <20%, 17.21 (95% CI, 5.44–54.47); and 20% to <35%, 61.80 (95% CI, 26.77–142.64); Supplementary Fig. S9A]. However, we did not observe a similar trend for the other outcomes, and point estimates did not increase as the VAFs increased (Supplementary Figs. S9B–S9F and S10).

## Discussion

Our study identified 1,157 *TP53*-CHIP carriers and provided the largest evidence to date (to the best of our knowledge) on mutation characteristics and clinical management. The prevalence of *TP53*-CHIP increased remarkably with age, regardless of the health status, and *TP53*-CHIP was associated with a wide range of medical conditions.

In this study, we observed an association between smoking habits and *TP53*-CHIP. Smoking habits might be a risk factor for *TP53*-CHIP, as shown for *ASXL1* (6). In the present study, drinking habits among individuals with *ALDH2* rs671 Lys+ were associated with *TP53*-CHIP, resulting from a gene–environment interaction. A previous genomic evaluation of esophageal cancer reveals enrichment of T>C *TP53* mutations in alcohol drinkers when compared with nondrinkers, suggesting that alcohol-derived acetaldehyde may contribute to *TP53* mutation (16). Similarly, the present study indicated that alcohol-derived acetaldehyde may contribute to the increased risk of *TP53*-CHIP. *ALDH2* rs671 is an East Asian–specific common variant (12). The mutation landscape among cancer genomes across various cancer types differs between East Asian and Western patients, with higher *TP53* mutation frequencies observed in the former (17). The results of the present study indicate that the landscape of CHIP could also vary by region and population because of gene–environment interactions, providing novel insights into the mechanisms underlying mutation evolution.

We did not observe any difference in blood cell count abnormalities between *TP53*-CHIP carriers and noncarriers, noting no correlation between blood cell counts and VAFs. These findings support the concept that *TP53*-CHIP is a pre-disease condition rather than myeloid neoplasms. The present study showed that *TP53*-CHIP was associated with a significantly increased risk of myeloid neoplasm mortality. Besides, HRs of myeloid neoplasm mortality remarkably increased as the VAFs of *TP53*-CHIP increased. A previous study reported that the time to develop myeloid neoplasms is inversely correlated with higher VAFs of *TP53*-CHIP (5). The present study confirmed the increasing HRs of myeloid neoplasm mortality through large-scale and high-resolution evaluation, followed by long-term follow-up data. However, no association was found between the degree of clonal expansion and risk of other outcomes, including lymphoid neoplasms. Beyond myeloid neoplasms, the presence of *TP53*-CHIP itself may be clinically relevant regardless of VAF. Although further evaluation is needed to determine the significance of small clones, it may be important to consider not only *TP53*-CHIP cases with higher VAFs but also those with lower VAFs in the clinical settings.

Interestingly, individuals with both environmental factors (acetaldehyde exposure or smoking) and *TP53*-CHIP showed a remarkably higher risk for myeloid neoplasms or respiratory disease mortality, accompanied by excess risks. Although these environmental factors were risk factors for *TP53*-CHIP, we observed significant interactions between them and *TP53*-CHIP for disease-specific mortality that operated only when *TP53*-CHIP was present in the mediation analyses. These indicate that *TP53*-CHIP acquired through a process distinct from these environmental factors boosted the risk of disease-specific mortality. In mouse models, aldehyde-driven chronic endogenous DNA damage drives hematopoietic stem and progenitor cell aging in a p53-dependent manner (18). Taken together with our results, this suggests that the coexistence of *TP53*-CHIP in individuals prone to acetaldehyde exposure significantly accelerates the risk of myeloid neoplasms. Inflammation, directly regulated by IL-6 signaling, is involved in the development of several diseases (15). With regard to respiratory disease, *TET2* knockout in blood cells influences pulmonary inflammation and exacerbates the development of emphysema in mice (19). p53 regulates Tet2 stability through autophagic degradation pathways (20, 21). Indeed, individuals with both a genetic proxy for IL-6 inhibition and *TP53*-CHIP tended to be at a lower risk of respiratory disease mortality than *TP53*-CHIP alone in our present study. Thus, *TP53*-CHIP may also play a key role in regulating inflammation among respiratory organs. From another perspective, when individuals with corresponding environmental factors have *TP53*-CHIP, they should be more strictly followed for myeloid neoplasms or respiratory disease. These findings contribute to the current understanding of pathogenesis as well as to personalize risk stratification.

This study has some limitations. First, only participants from hospital-based biobanks were included, which may underrepresent the healthy population. However, CHIP occurs in older individuals and those with comorbidities (1). As these individuals visit certain hospitals, the population evaluated in our study, which includes many general hospitals (9–11), does not seem to deviate significantly from the general population. Second, with regard to environmental risk factors, unmeasured confounding factors might bias the results because of the retrospective nature of our evaluation. Thus, prospective studies are warranted. Third, our evaluation was based on the East Asian population, which may limit the extent to which the results of this study are relevant to other populations. Further additional evaluation is warranted.

In conclusion, we performed the largest evaluation of *TP53*-CHIP to the best of our knowledge, which plays an important role but is relatively infrequent in CHIP. This evaluation highlighted the clinical significance of *TP53*-CHIP. The current findings provide a basis for further research focused on elucidating pathogenesis and improving clinical management.

## Methods

### Study Participants and Samples

A total of 140,597 individuals without hematologic neoplasms were included in this study from BioBank Japan, which is a multi-institutional hospital registry that collected peripheral blood samples and clinical information from all over Japan between 2003 and 2018; the sampling is representative of the general patient population in Japan (9–11). DNA was extracted form peripheral whole blood cells at the commercial laboratories according to each laboratory’s standard procedures and stored in the DNA bank at 4°C (9–11). All participants provided written informed consent. This study was approved by the ethics committees of the Institute of Medical Sciences, the University of Tokyo, and the RIKEN Center for Integrative Medical Sciences.

### Sequencing and Bioinformatic Analysis

CHIP was evaluated for all coding regions and 2 bp flanking intronic sequences of *TP53* according to the Consensus CDS, release 15 (CCDS45605, CCDS45606, and CCDS11118; 1,311 bp). The pooled DNA libraries were sequenced using 2 × 150 bp paired-end reads on HiSeq 2500 or NovaSeq 6000 (Illumina, Inc.). We used raw sequencing data of *TP53* from our studies (22–27) that assess germline variants for disease risk. Sequencing reads were aligned to the human reference genome (hg19) using Burrows–Wheeler Aligner. Putative somatic mutations were identified using Mutect2 (GATK; RRID: SCR_001876; v4.3.0.0) and Samtools (RRID: SCR_002105) mpileup. Considering that a previous study (7) reported that the VAFs of almost all mutations are less than 35% and that VAFs of 35% or more include germline variants, we excluded mutations with VAFs of 35% or more to eliminate the possibility of germline variants thoroughly, although it is possible to miss some carriers. We cannot rule out tiny amounts of tumor-derived DNA contamination although the ratio of DNA of blood cells to tumor-derived DNA is hugely different. Tumor DNA–contaminated samples likely would have very low VAFs. To eliminate such cases, we also excluded mutations with VAFs of less than 2%. Therefore, we focused on mutations with VAFs of 2% or more and less than 35%, which met a total sequence read count of 100 or more and an alternative sequence read count of 10 or more. We visualized the relationship between VAF and sequence reads as well as VAF and mapping quality and curated all putative mutations. We removed mutations as germline variants, in which the number of individuals with VAFs of 2% or more and less than 35% was less than the number of individuals with VAFs of 35% or more. In addition, those with a frequency of more than 1% in the total study population were excluded as suspected artifacts. To remove strand bias, we manually checked the Integrative Genomics Viewer for all mutations that met the following conditions among the Fisher exact test between forward and reverse strands for the reference or alternate allele: OR <0.5 or >2 and Phred-scaled *P* > 30. Excluding routinely all mutations registered in ClinVar (RRID: SCR_006169) may overexclude important somatic mutations because some of them are also oncogenic mutations in the somatic context (28). Instead, we checked that the mutations finally identified were not common variants in gnomAD (RRID: SCR_014964). Furthermore, we performed a sensitivity analysis, excluding those with registered mutations in ClinVar (RRID: SCR_006169) as pathogenic/likely pathogenic and VAFs of 25% or more. We confirmed that this targeted sequencing was comparable with high-coverage and PCR-free whole-genome sequencing for high sensitivity and accuracy (Supplementary Fig. S11A and S11B). The amino acid positions of the detected mutations were determined according to the CCDS11118.

### ddPCR

To validate detected mutations, we conducted ddPCR for the top five distinct mutations. Predesigned probes were purchased from Bio-Rad. Catalog numbers for the probe mix were dHsaMDV2010109, dHsaMDV2510536, dHsaMDV2010127, dHsaMDV2010105, and dHsaMDV2010107. We mixed 20 ng of DNA, enzymes [ddPCR Supermix for Probes (no dUTP), Bio-Rad], and the probe mix, followed by droplet generation and PCR amplification according to the manufacturer’s protocol. The annealing temperature was set at 55°C. We measured amplified droplets using the QX200 system and QuantaSoft 1.7. Bland–Altman plots of differences versus means were used to evaluate the presence of fixed and/or proportional bias (29, 30). The mean of difference of VAF was calculated to assess the fixed bias between targeted sequencing and ddPCR. A regression analysis between the differences of VAF and the corresponding means of VAF was performed, and a slope different from zero was used to indicate the presence of proportional bias. Confidence intervals of 95% were estimated by bootstrapping the data 1,000 times. The intraclass correlation coefficient was also calculated to examine the agreement between targeted sequencing and ddPCR.

### Statistical Analysis

We described the prevalence of *TP53*-CHIP and evaluated the characteristics of *TP53*-CHIP carriers and noncarriers among the study participants. We also estimated the ORs and their CIs to evaluate the impact of environmental factors (smoking and drinking habits) on the existence of *TP53*-CHIP using a logistic regression model adjusted for potential confounders according to the modified disjunctive cause criterion (31): age, sex, drinking habits (ever, never, and missing), alcohol consumption (<23 g/day, 23 to <46 g/day, ≥46 g/day, and missing), smoking habits (ever, never, and missing), Brinkman index (<400, 400 to <800, 800 to <1,200, ≥1,200, and missing), body mass index (<23, 23 to <25, ≥25, and missing), and comorbidities (hyperlipidemia, hypertension, diabetes, and cancer). Furthermore, we compared the blood cell counts between *TP53*-CHIP noncarriers and carriers. Data on blood cell counts measured within 30 days of blood sample collection were obtained. Abnormalities in blood cell counts were defined following the definitions of a previous study (32): (i) white blood cells (/µL) ≥10,000 or <3,000; (ii) hemoglobin (g/dL) ≥16.5 (male), ≥16 (female), or <10; (iii) hematocrit (%) ≥50; and (iv) platelets (×10^4^/µL) ≥50 or <10. We compared the proportions of abnormalities in blood cell counts in *TP53*-CHIP carriers and noncarriers using the *χ*^2^ test. The associations between VAFs of *TP53*-CHIP and blood cell counts were also evaluated using linear regression.

A follow-up survival survey was conducted based on the date of the last hospital visit, resident cards, and vital statistics (9, 11). Overall survival was defined as the period from the date of registration to all-cause mortality or the last follow-up day. For the causes of death, we included diagnoses within the ICD-10 major categories, with 10 or more carriers of *TP53*-CHIP. The information of the ICD-10 was collected from the vital statistics by the Statistics and Information Department of the Ministry of Health, Labour, and Welfare, Japan (9, 11). We independently defined the causes of death according to the ICD-10 for this study. We evaluated the association between *TP53*-CHIP and overall survival probability using the Kaplan–Meier method and compared the results between carriers and noncarriers using the log-rank test. The cumulative incidence of disease-specific mortality was visualized by considering other causes of death as competing risks and compared using the Gray test. The cause-specific hazard model, which is suited for studying the etiology of diseases, was applied to evaluate disease-specific mortality (33). To estimate the HRs and their 95% CIs, we applied the Cox proportional hazards model for multivariable analysis adjusted for potential confounders according to the modified disjunctive cause criterion (31): age, sex, drinking habits (ever, never, and missing), alcohol consumption (<23 g/day, 23 to <46 g/day, ≥46 g/day, and missing), smoking habits (ever, never, and missing), Brinkman index (<400, 400 to <800, 800 to <1,200, ≥1,200, and missing), body mass index (<23, 23 to <25, ≥25, and missing), and comorbidities (hyperlipidemia, hypertension, diabetes, and cancer). Interactions between *TP53*-CHIP and environmental factors for disease-specific mortality were also estimated as an additive interaction, measured as the relative excess risk due to interaction (34), and as a multiplicative interaction, measured with the use of interaction terms. Furthermore, to describe the relationships among environmental factors, *TP53*-CHIP, and disease-specific mortality, we performed a mediation analysis by using the Stata command *med4way* (35). Mediation analysis is a statistical method for understanding the impact of an exposure on an outcome through the influence of a third variable, an intermediate factor. Briefly, it helps to elucidate the mechanism by separating the direct and indirect effects of an exposure on an outcome.

All statistical tests were two-sided, and statistical significance was set at *P* < 0.05. Benjamini–Hochberg multiple test correction was conducted where appropriate. All statistical analyses were performed using Stata version 18.0 (Stata Corp.) and R version 3.5.2 (R Foundation for Statistical Computing).

### Data Availability

Primary sequencing data used in this study are available from National Bioscience Database Center (https://humandbs.dbcls.jp) with accession number JGAS000782 under National Bioscience Database Center Data Sharing Policy (controlled access data type-1).

## Supplementary Material

Table S1 Characteristics of study participants

Table S2 Detected mutations

Table S3 Association of environmental factors with TP53-CHIP

Table S4 Causes of death for evaluation of disease-specific mortality

Table S5 Association of TP53-CHIP with each outcome among all models

Table S6 Stratified analyses by environmental factors

Figure S1 Bland-Altman plots for targeted sequencing and ddPCR

Figure S2 Prevalence of TP53-CHIP

Figure S3 Proportion of mutation patterns observed in TP53 according to alcohol consumption among individuals with rs671 Lys+

Figure S4 Probability of overall survival and cumulative incidence of disease-specific mortality

Figure S5 Sensitivity analysis of disease-specific mortality considering ALDH2 rs671

Figure S6 Sensitivity analysis excluded TP53-CHIP carriers with mutations registered in ClinVar as pathogenic/likely pathogenic and VAFs of 25% or more

Figure S7 Stratified by TP53-CHIP carrier status and genetically determined impairment in interleukin (IL)-6 signaling among individuals with smoking habits

Figure S8 Mediation analyses

Figure S9 Clinical impact of TP53-CHIP by VAF

Figure S10 Probability of overall survival by VAF

Figure S11 Performance of mutation call
