# Supplementary material for: Clinical Significance of TP53-Mutant Clonal Hematopoiesis Across Diseases
Source: Blood Cancer Discov. 2025 Jun 17;6(4):298–306. doi: 10.1158/2643-3230.BCD-24-0355 (PMC12209765; doi:10.1158/2643-3230.BCD-24-0355)
Supplement: Table S1 — Characteristics of study participants [file bcd-24-0355_table_s1_suppst1.pdf]

Table S1. Characteristics of study participants

(A) Overall participants

	N = 140,597
Age, mean (SD)	65.48 (12.49)
Sex	
Male (%)	78,411 (55.77)
Female (%)	62,187 (44.23)
Body mass index (%)	
<23	72,017 (51.22)
23 to <25	29,301 (20.84)
≥25	34,950 (24.86)
Missing	4,329 (3.08)
Comorbidities (%)	
Cancer	72,313 (51.43)
Hypertension	37,240 (26.49)
Hyperlipidemia	40,979 (29.15)
Diabetes	34,588 (24.60)
Smoking habits (%)	
Never	65,238 (46.40)
Ever	72,623 (52.36)
Missing	1,736 (1.23)
Drinking habits (%)	
Never	65,899 (46.87)
Ever	72,600 (51.64)
Missing	2,098 (1.49)

(B) Follow-up survival survey participants

	N = 81,462
Age, mean (SD)	65.55 (11.62)
Sex	
Male (%)	46,926 (57.60)
Female (%)	34,536 (42.40)
Body mass index (%)	
<23	40,982 (50.31)
23 to <25	17,086 (20.97)
≥25	20,591 (25.28)
Missing	2,803 (3.44)
Comorbidities (%)	
Cancer	39,131 (48.04)
Hypertension	21,584 (26.50)
Hyperlipidemia	26,708 (32.79)
Diabetes	23,096 (23.35)
Smoking habits (%)	
Never	36,717 (45.07)
Ever	43,733 (53.69)
Missing	1,012 (1.24)
Drinking habits (%)	
Never	38,203 (46.90)
Ever	42,111 (51.69)
Missing	1,148 (1.41)
Follow-up time, median (IQR)	9.95 (6.07–11.66)
Age of death, mean (SD)	74.17 (11.32)
[a]Cause of death, number	
Non-hematological neoplasms	13,256
Cardiovascular disease	5,645
Respiratory disease	3,405
Lymphoid neoplasms	199
Myeloid neoplasms	162

[a]The causes of death were defined according to the International Classification of Diseases, Tenth Revision (ICD-10): non-hematological neoplasms (C00–C80), cardiovascular disease (I00–I99), respiratory disease (J00–J22), lymphoid neoplasms (C81–C91), and myeloid neoplasms (C92–C95, D46).
