# Supplementary material for: Clinical Significance of TP53-Mutant Clonal Hematopoiesis Across Diseases
Source: Blood Cancer Discov. 2025 Jun 17;6(4):298–306. doi: 10.1158/2643-3230.BCD-24-0355 (PMC12209765; doi:10.1158/2643-3230.BCD-24-0355)
Supplement: Table S3 — Association of environmental factors with TP53-CHIP [file bcd-24-0355_table_s3_suppst3.pdf]

Table S3. Association of environmental factors with *TP53*-CHIP

(A) Overall

	OR	95% CI	P value
Never-smoker	1.00	(1.00–1.00)	
Ever-smoker (BI: <400)	1.29	(1.05–1.59)	
Ever-smoker (BI: 400 to <800)	1.37	(1.12–1.67)	for trend **2.95×10^{-5}**
Ever-smoker (BI: 800 to <1200)	1.25	(1.01–1.55)	
Ever-smoker (BI: ≥1200)	1.62	(1.32–1.98)	
Ever-smoker (all)	1.37	(1.18–1.59)	**3.43×10^{-5}**
Never-drinker	1.00	(1.00–1.00)	
Ever-drinker (gpd: <23)	1.07	(0.91–1.25)	for trend 0.531
Ever-drinker (gpd: 23 to <46)	1.10	(0.84–1.44)	
Ever-drinker (gpd: ≥46)	1.03	(0.75–1.43)	
Ever-drinker (all)	1.11	(0.97–1.27)	0.128

(B) Among individuals with rs671 Lys+

	OR	95% CI	P value
Never-drinker	1.00	(1.00–1.00)	
Ever-drinker (gpd: <23)	1.30	(1.02–1.66)	for trend **3.11×10^{-7}**
Ever-drinker (gpd: 23 to <46)	2.23	(1.43–3.46)	
Ever-drinker (gpd: ≥46)	2.68	(1.63–4.40)	
Ever-drinker (all)	1.43	(1.19–1.73)	**1.75×10^{-4}**

Odds ratios (ORs) and their 95% confidence intervals (CIs) were estimated using logistic regression models; for drinking habits: adjusted for age, sex, smoking habits, Brinkman index, body mass index, and comorbidities (hyperlipidemia, hypertension, diabetes, and cancer), for smoking habits: adjusted for age, sex, drinking habits, alcohol consumption, body mass index, and comorbidities (hyperlipidemia, hypertension, diabetes, and cancer). Bold indicates $q < 0.05$ after Benjamini–Hochberg multiple test correction.

Abbreviations: BI, Brinkman index; gpd, g/day.
