# Supplementary material for: Clinical Significance of TP53-Mutant Clonal Hematopoiesis Across Diseases
Source: Blood Cancer Discov. 2025 Jun 17;6(4):298–306. doi: 10.1158/2643-3230.BCD-24-0355 (PMC12209765; doi:10.1158/2643-3230.BCD-24-0355)
Supplement: Table S4 — Causes of death for evaluation of disease-specific mortality [file bcd-24-0355_table_s4_suppst4.pdf]

Table S4. Causes of death for evaluation of disease-specific mortality

Disease	ICD-10 code
Non-hematological neoplasms	C00–C80
Gastric cancer	C16
Colorectal cancer	C18–C20
Liver cancer	C22
Pancreatic cancer	C25
Lung cancer	C34
Breast cancer	C50
Prostate cancer	C61
Lymphoid neoplasms	C81–C91
Myeloid neoplasms	C92–C95, D46
Cardiovascular disease	I00–I99
Ischemic heart disease	I20–I25
Stroke	I60–69
Respiratory disease	J00–J99
Respiratory tract infection	J00–J22
Chronic lower respiratory tract disease	J40–J47
Interstitial pneumonia	J84
