# Supplementary material for: Clinical Significance of TP53-Mutant Clonal Hematopoiesis Across Diseases
Source: Blood Cancer Discov. 2025 Jun 17;6(4):298–306. doi: 10.1158/2643-3230.BCD-24-0355 (PMC12209765; doi:10.1158/2643-3230.BCD-24-0355)
Supplement: Figure S1 — Bland-Altman plots for targeted sequencing and ddPCR [file bcd-24-0355_figure_s1_suppsf1.pdf]

Figure S1. Bland-Altman plots for targeted sequencing and ddPCR

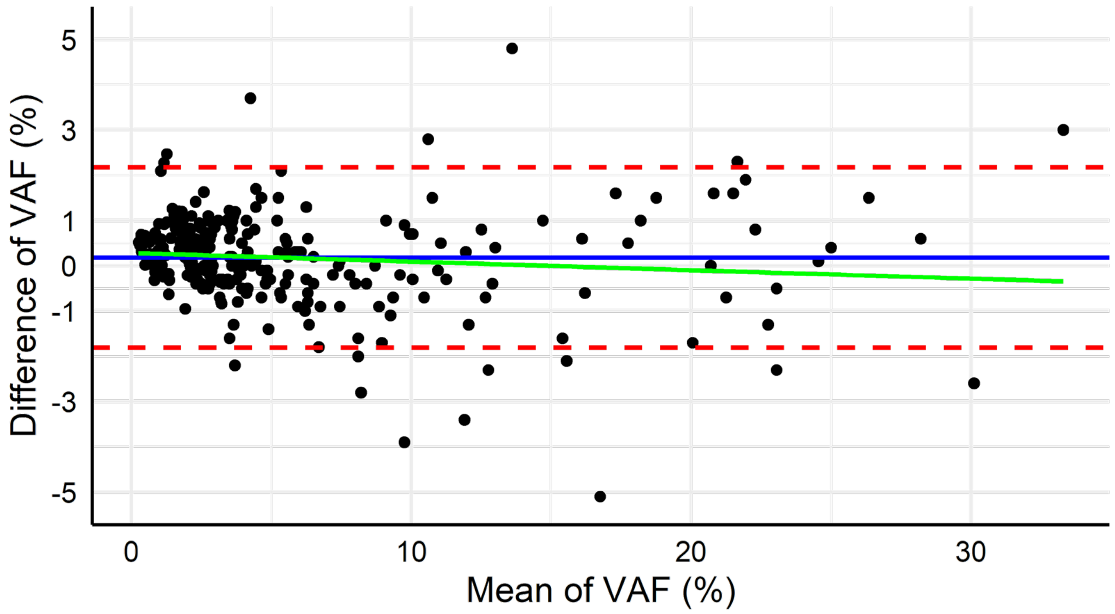

Difference of VAF: VAF in targeted sequencing - VAF in ddPCR

Mean of VAF: average VAF calculated from VAF in targeted sequencing and VAF in ddPCR

Green line: regression line between the differences of VAF and the corresponding means of VAF

Blue line: mean of difference

Red dashed line: 95% limits of agreement

The mean of the difference: 0.18% [95% confidence interval, 0.06% to 0.30%]

The slope of the regression line: -0.02 [95% confidence interval, -0.05 to 0.01]

(95% confidence intervals were estimated by bootstrapping the data 1,000 times)
