# Supplementary material for: Clinical Significance of TP53-Mutant Clonal Hematopoiesis Across Diseases
Source: Blood Cancer Discov. 2025 Jun 17;6(4):298–306. doi: 10.1158/2643-3230.BCD-24-0355 (PMC12209765; doi:10.1158/2643-3230.BCD-24-0355)
Supplement: Figure S2 — Prevalence of TP53-CHIP [file bcd-24-0355_figure_s2_suppsf2.pdf]

Figure S2. Prevalence of *TP53*-CHIP

(A)

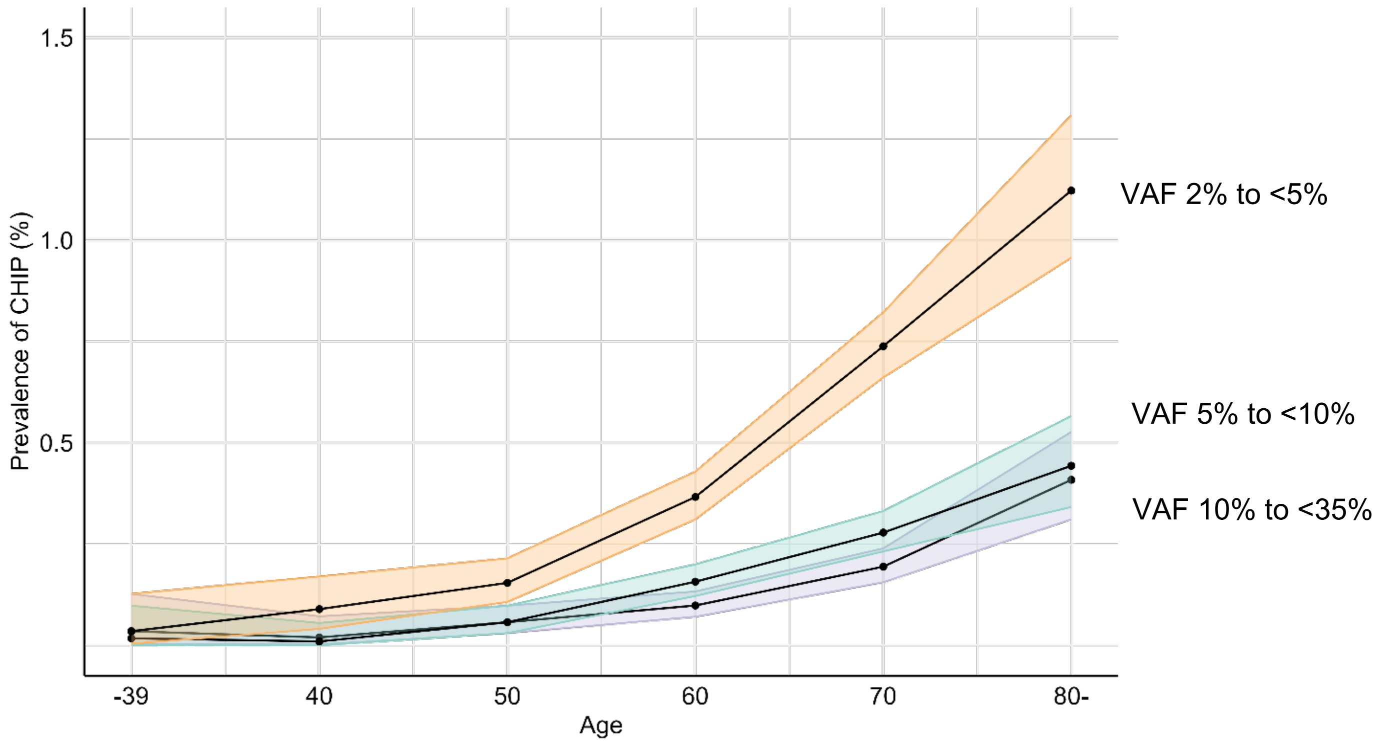

(B)

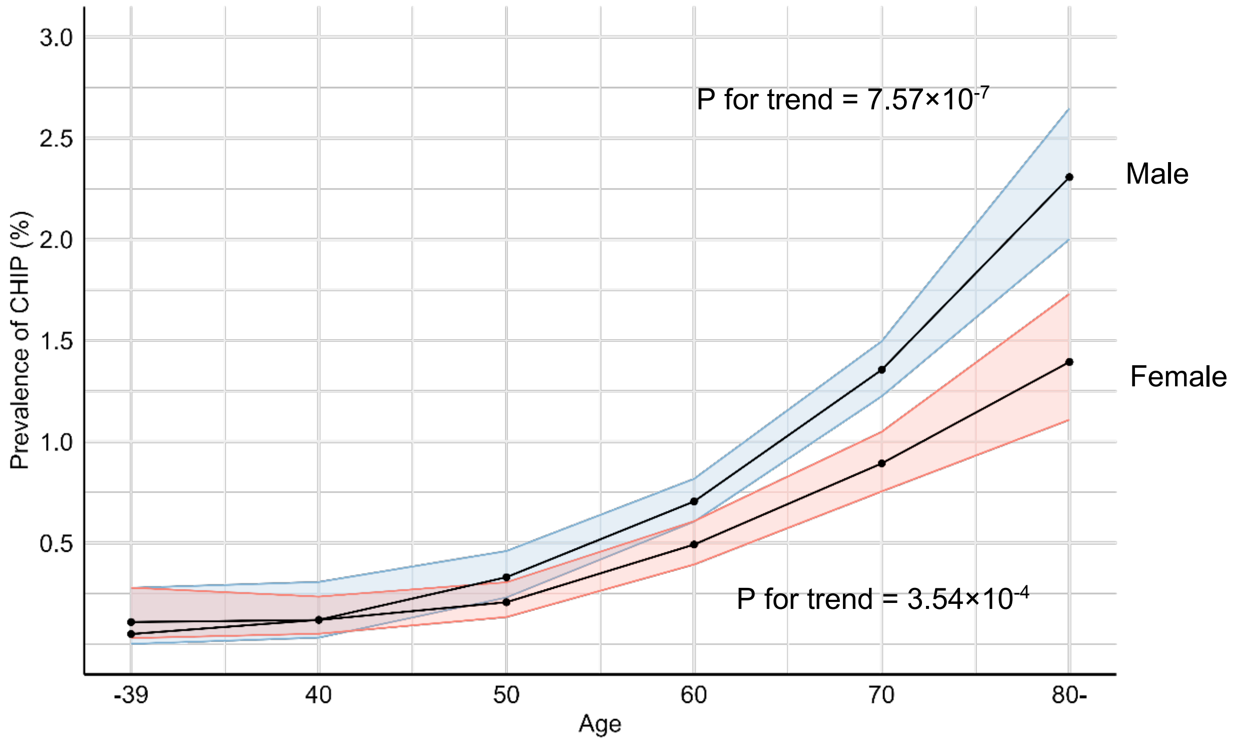

(C)

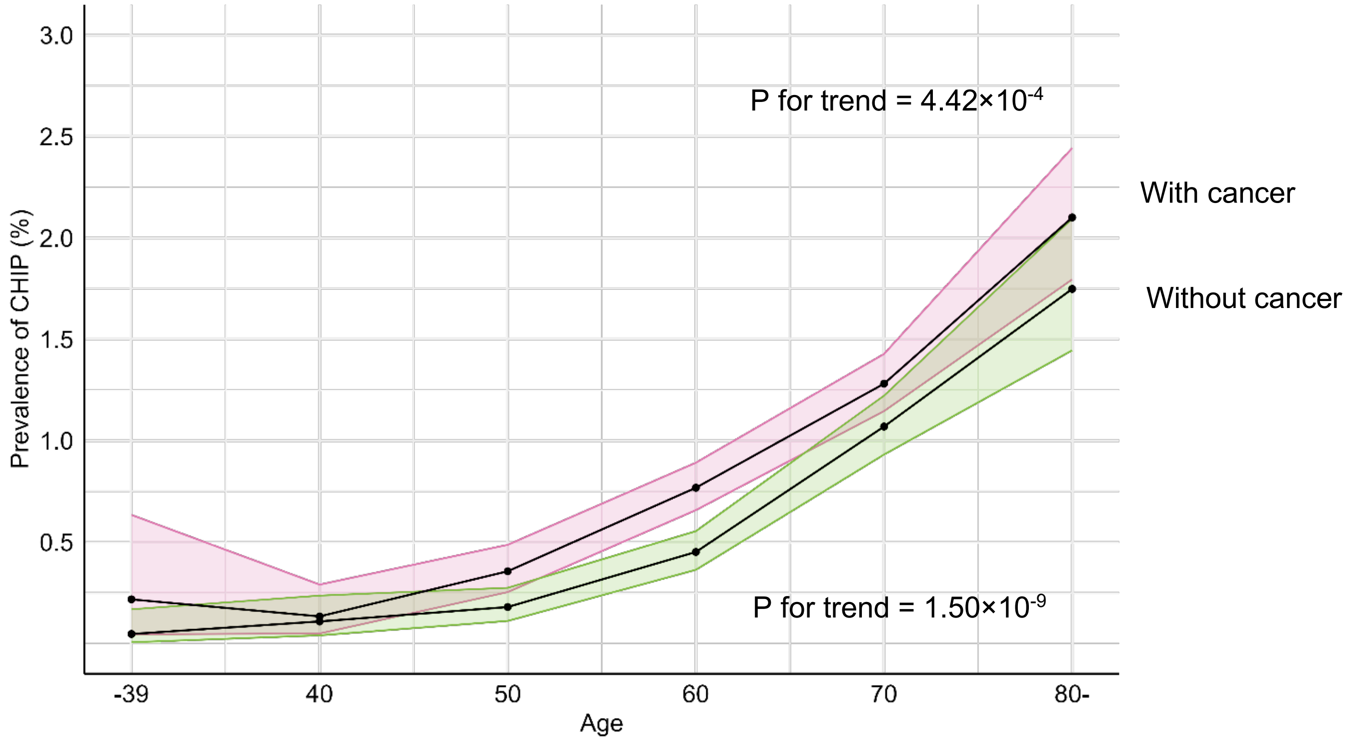

Prevalence of (A) *TP53*-CHIP by VAF (2% to <5%: 686 carriers, 5% to <10%: 265 carriers, 10% to <35%: 206 carriers), (B) *TP53*-CHIP with VAF 2% to <35% by sex (male: 806 carriers, female: 351 carriers), and (C) *TP53*-CHIP with VAF 2% to <35% by cancer status (with cancer: 708 carriers, without cancer: 449 carriers). The shaded areas indicate the 95% confidence intervals estimated using the Clopper–Pearson method. Trends were evaluated using the Cochran–Armitage test.
