# Supplementary material for: Clinical Significance of TP53-Mutant Clonal Hematopoiesis Across Diseases
Source: Blood Cancer Discov. 2025 Jun 17;6(4):298–306. doi: 10.1158/2643-3230.BCD-24-0355 (PMC12209765; doi:10.1158/2643-3230.BCD-24-0355)
Supplement: Figure S3 — Proportion of mutation patterns observed in TP53 according to alcohol consumption among individuals with rs671 Lys+ [file bcd-24-0355_figure_s3_suppsf3.pdf]

**Figure S3. Proportion of mutation patterns observed in *TP53* according to alcohol consumption among individuals with rs671 Lys+**

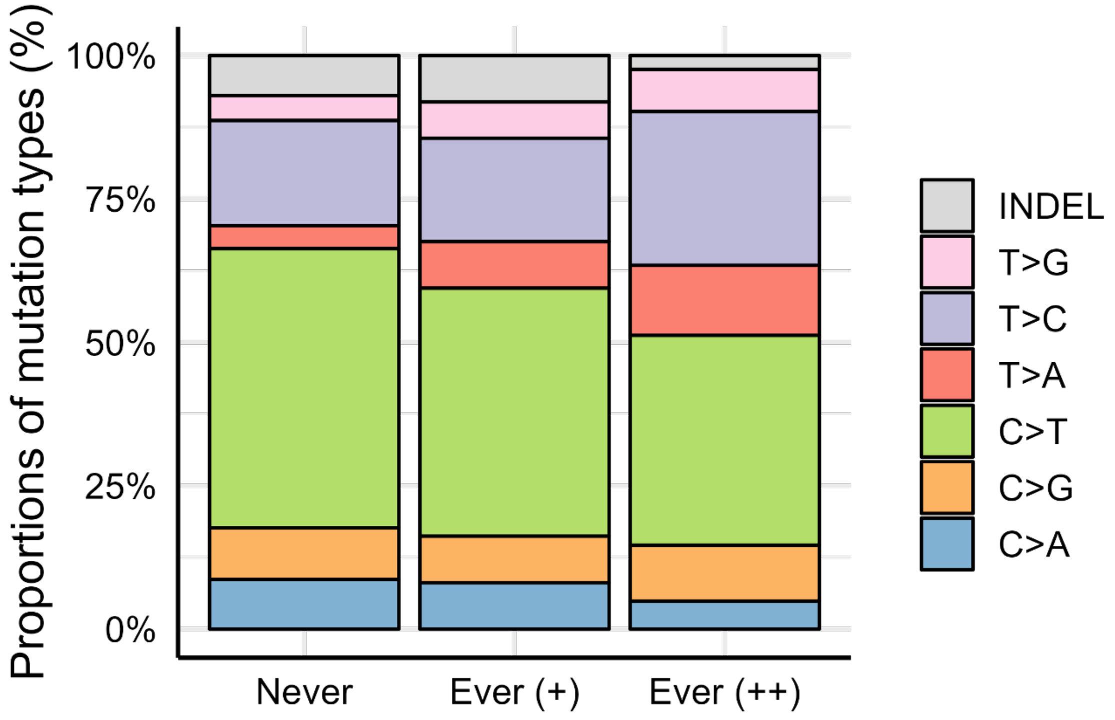


Ever (+): 23 to <46 g/day, Ever (++): ≥46 g/day

After stratifying alcohol consumption among individuals with rs671 Lys+, the proportion of T>C observed in *TP53* was higher with alcohol consumption (Never: 18.33% in 300 mutations, Ever (+): 18.02% in 111 mutations, and Ever (++): 26.83% in 41 mutations), although the difference was not statistically significant (P [Chi-square test] = 0.467).
