# Supplementary material for: Clinical Significance of TP53-Mutant Clonal Hematopoiesis Across Diseases
Source: Blood Cancer Discov. 2025 Jun 17;6(4):298–306. doi: 10.1158/2643-3230.BCD-24-0355 (PMC12209765; doi:10.1158/2643-3230.BCD-24-0355)
Supplement: Figure S4 — Probability of overall survival and cumulative incidence of disease-specific mortality [file bcd-24-0355_figure_s4_suppsf4.pdf]

Figure S4. Probability of overall survival and cumulative incidence of disease-specific mortality

(A)

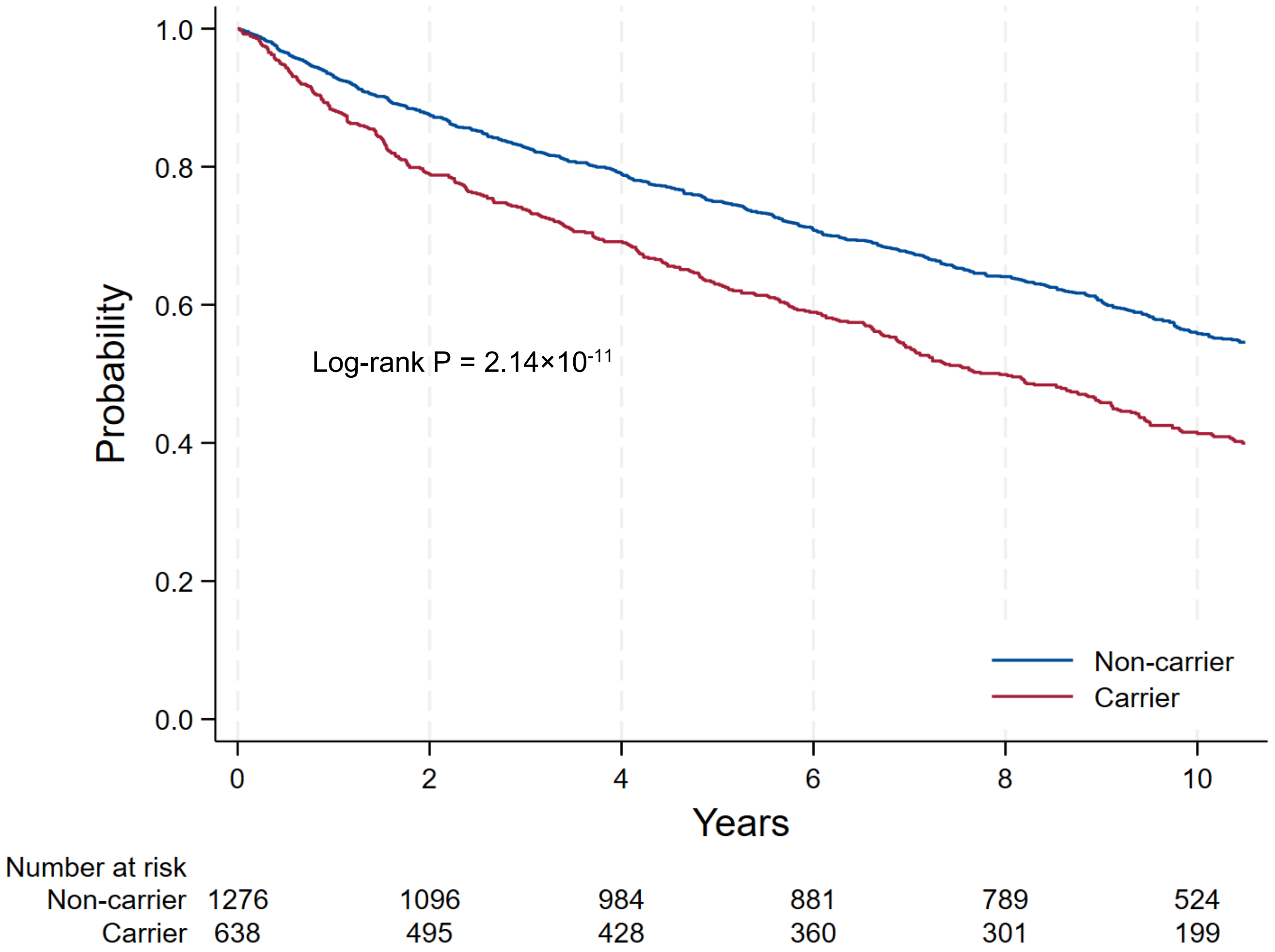

(B)

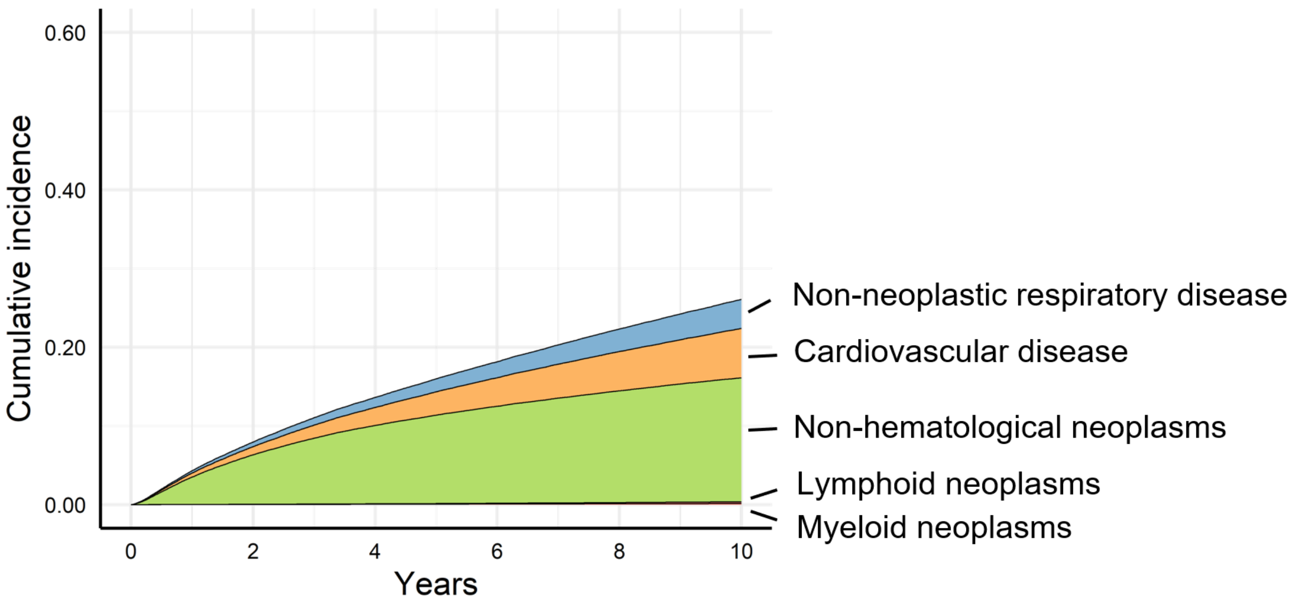

(C)

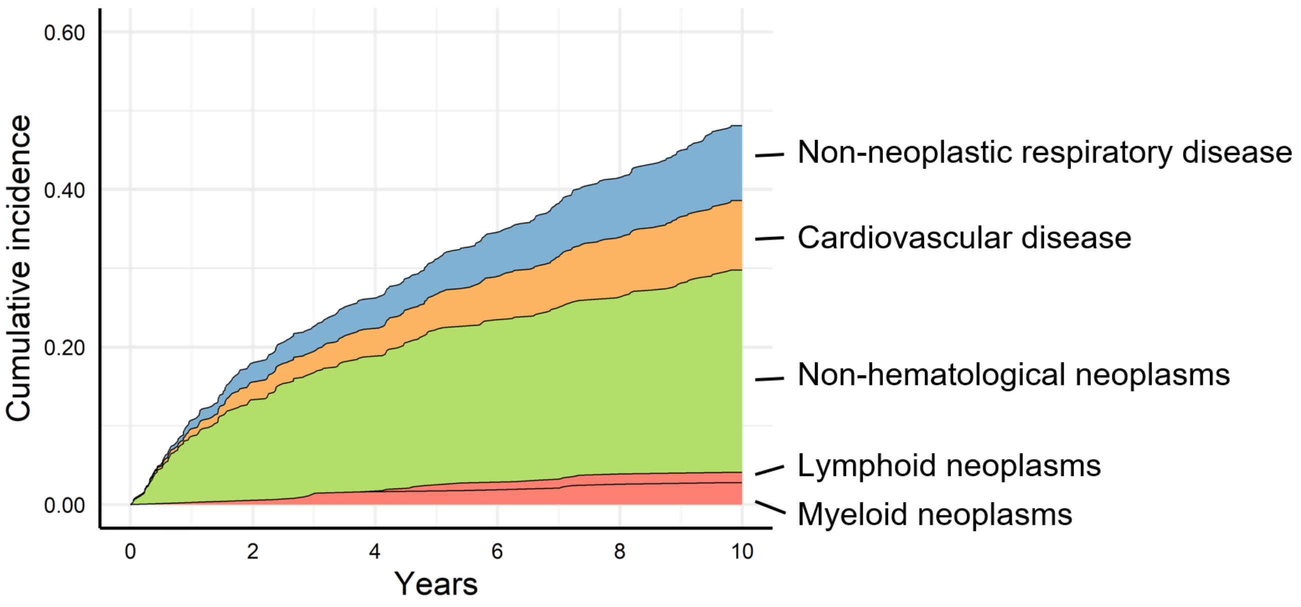

(A) The probability of overall survival was estimated using the Kaplan–Meier method and compared using the log-rank test. Non-carriers were selected using 1:2 exact matching based on age and sex (mean age [standard deviation]: 72.50 [8.83]; proportion of males: 71.63% in both carriers and non-carriers). (B)(C) The cumulative incidence of disease-specific mortality among individuals with or without *TP53*-CHIP were visualized by considering other causes of death as competing risks ([B] non-carrier, [C] carrier).
