# Supplementary material for: Clinical Significance of TP53-Mutant Clonal Hematopoiesis Across Diseases
Source: Blood Cancer Discov. 2025 Jun 17;6(4):298–306. doi: 10.1158/2643-3230.BCD-24-0355 (PMC12209765; doi:10.1158/2643-3230.BCD-24-0355)
Supplement: Figure S5 — Sensitivity analysis of disease-specific mortality considering ALDH2 rs671 [file bcd-24-0355_figure_s5_suppsf5.pdf]

Figure S5. Sensitivity analysis of disease-specific mortality considering *ALDH2* rs671

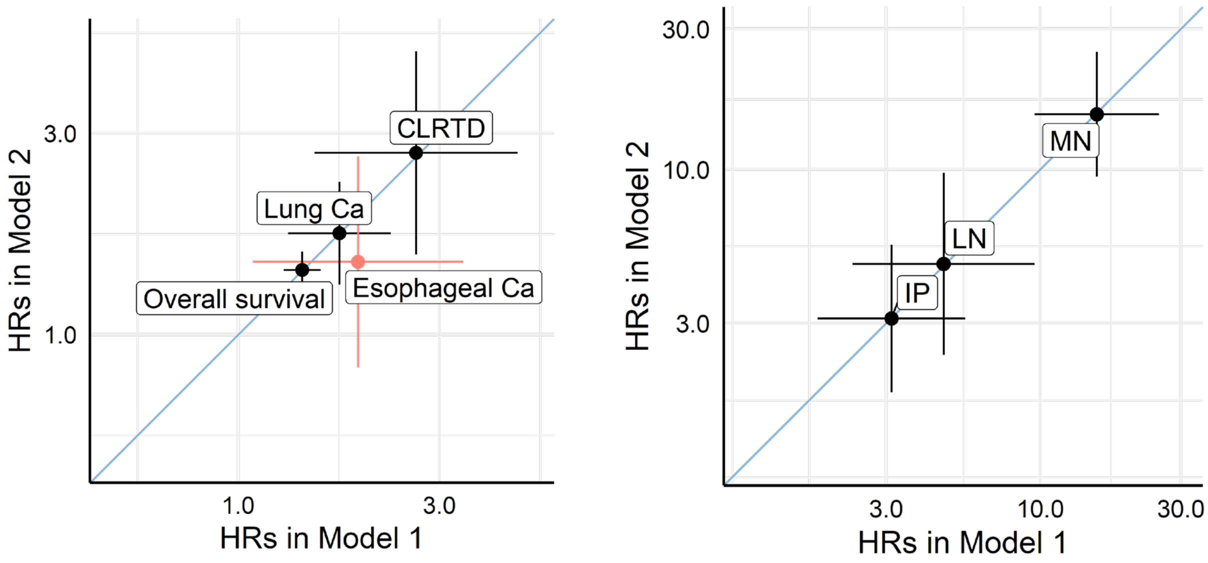

Hazard ratios (HRs) and their 95% confidence intervals (CIs) were estimated using Cox proportional hazard models of disease-specific mortality.

Model 1: adjusted for age, sex, drinking habits, alcohol consumption, smoking habits, Brinkman index, body mass index, and comorbidities (hyperlipidemia, hypertension, diabetes, and cancer)

Model 2: adjusted for age, sex, drinking habits, alcohol consumption, smoking habits, Brinkman index, body mass index, and comorbidities (hyperlipidemia, hypertension, diabetes, and cancer), and *ALDH2*

Abbreviations: CLRTD, chronic lower respiratory tract disease; IP, interstitial pneumonia; LN, lymphoid neoplasms; MN, myeloid neoplasms.
