# Supplementary material for: Clinical Significance of TP53-Mutant Clonal Hematopoiesis Across Diseases
Source: Blood Cancer Discov. 2025 Jun 17;6(4):298–306. doi: 10.1158/2643-3230.BCD-24-0355 (PMC12209765; doi:10.1158/2643-3230.BCD-24-0355)
Supplement: Figure S6 — Sensitivity analysis excluded TP53-CHIP carriers with mutations registered in ClinVar as pathogenic/likely pathogenic and VAFs of 25% or more [file bcd-24-0355_figure_s6_suppsf6.pdf]

Figure S6. Sensitivity analysis excluded *TP53*-CHIP carriers with mutations registered in ClinVar as pathogenic/likely pathogenic and VAFs of 25% or more

(A)

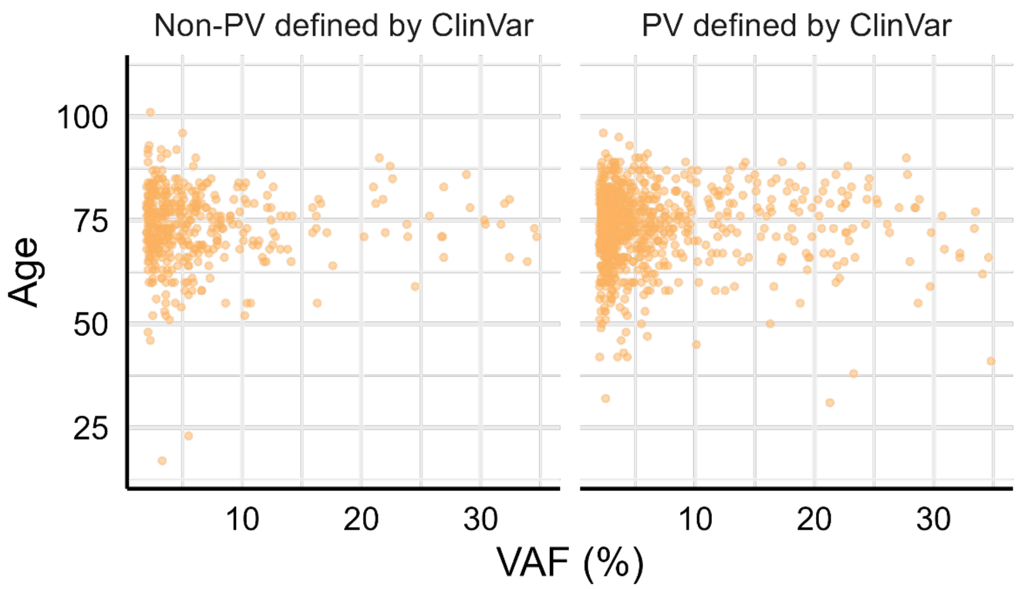

(B)

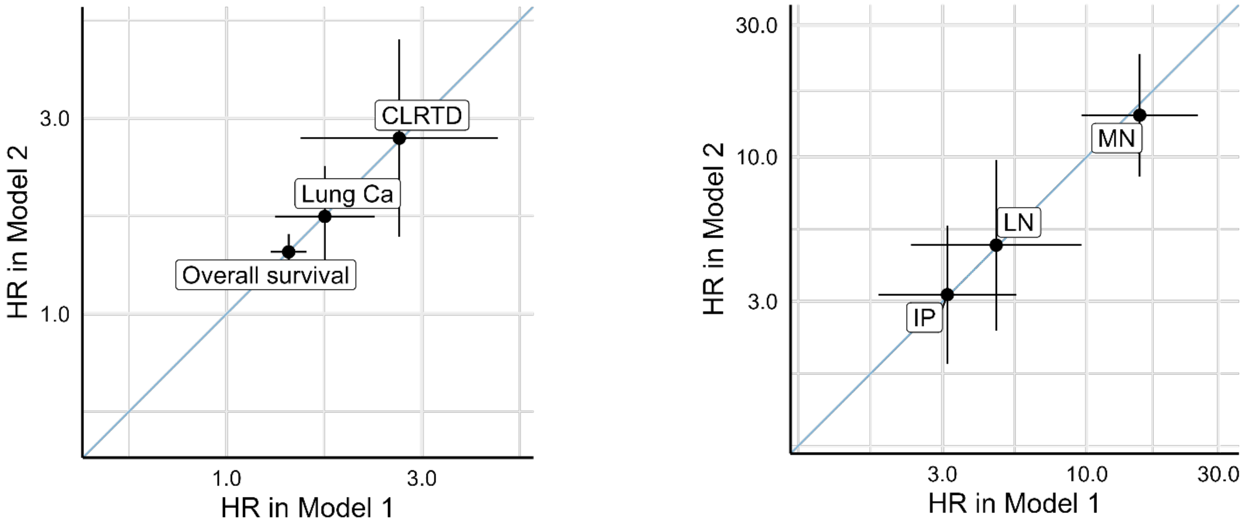

(A) Relationships between age and VAFs according to mutation status defined by ClinVar interpretations (2024-12-01) were described. Even among pathogenic/likely pathogenic variants (PVs) defined by ClinVar interpretations, most *TP53*-CHIP carriers were clustered in the lower VAF range and were middle-aged or older, indicating that misclassification of germline variants as CHIP was less likely. (B) Hazard ratios (HRs) and their 95% confidence intervals (CIs) were estimated using Cox proportional hazard models of disease-

specific mortality adjusted for age, sex, drinking habits, alcohol consumption, smoking habits, Brinkman index, body mass index, and comorbidities (hyperlipidemia, hypertension, diabetes, and cancer).

Model 1: conventional analysis

Model 2: excluded *TP53*-CHIP carriers with mutations registered in ClinVar as pathogenic/likely pathogenic and VAFs of 25% or more

Abbreviations: CLRTD, chronic lower respiratory tract disease; IP, interstitial pneumonia; LN, lymphoid neoplasms; MN, myeloid neoplasms.
