# Supplementary material for: Clinical Significance of TP53-Mutant Clonal Hematopoiesis Across Diseases
Source: Blood Cancer Discov. 2025 Jun 17;6(4):298–306. doi: 10.1158/2643-3230.BCD-24-0355 (PMC12209765; doi:10.1158/2643-3230.BCD-24-0355)
Supplement: Figure S7 — Stratified by TP53-CHIP carrier status and genetically determined impairment in interleukin (IL)-6 signaling among individuals with smoking habits [file bcd-24-0355_figure_s7_suppsf7.pdf]

Figure S7. Stratified by *TP53*-CHIP carrier status and genetically determined impairment in interleukin (IL)-6 signaling among individuals with smoking habits

(A)

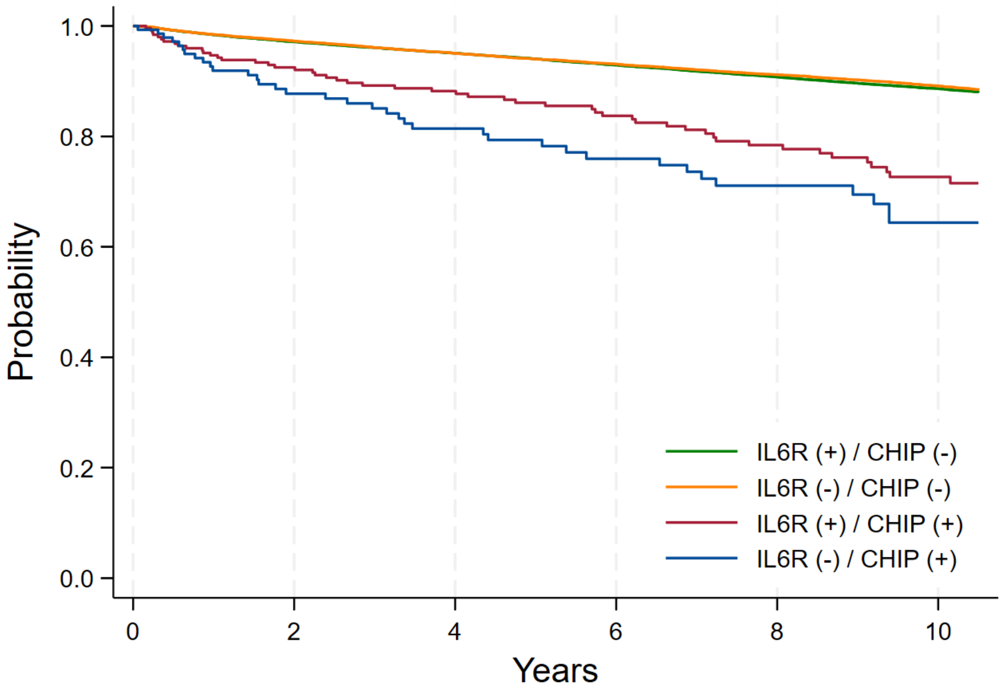

Number at risk						
IL6R (+) / CHIP (-)	26930	23545	21254	19359	17384	12207
IL6R (-) / CHIP (-)	15620	13664	12333	11233	10100	7096
IL6R (+) / CHIP (+)	256	201	174	137	108	69
IL6R (-) / CHIP (+)	145	102	83	66	54	27

(B)

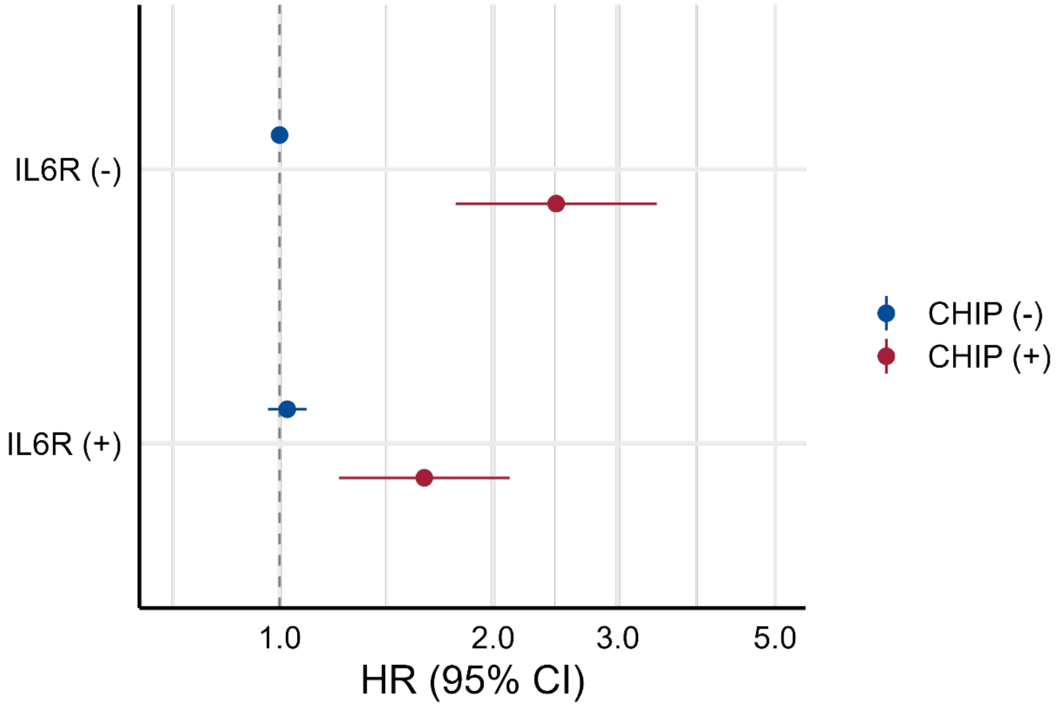

(A) The cause-specific survival from respiratory disease was estimated using the Kaplan–Meier method. (B) Hazard ratios (HRs) and their 95% confidence intervals (CIs) were estimated using Cox proportional hazard models of disease-specific mortality adjusted for age, sex, drinking habits, alcohol consumption, Brinkman index, body mass index, and comorbidities (hyperlipidemia, hypertension, diabetes, and cancer).

Smoking status was defined as ever-smoker.

IL6R (+) indicates individuals with *IL6R* rs2228145 Ala+, a genetic proxy for IL-6 inhibition(1).

Information of *IL6R* rs2228145 was obtained from publicly available database (National Bioscience Database Center, https://humandbs.dbcls.jp) with accession number JGAD000123 and JGAD000529.

Reference

1. Ferreira RC, Freitag DF, Cutler AJ, Howson JM, Rainbow DB, Smyth DJ*, et al.* Functional IL6R 358Ala allele impairs classical IL-6 receptor signaling and influences risk of diverse inflammatory diseases. PLoS Genet **2013**;9(4):e1003444 doi 10.1371/journal.pgen.1003444.
