# Supplementary material for: Clinical Significance of TP53-Mutant Clonal Hematopoiesis Across Diseases
Source: Blood Cancer Discov. 2025 Jun 17;6(4):298–306. doi: 10.1158/2643-3230.BCD-24-0355 (PMC12209765; doi:10.1158/2643-3230.BCD-24-0355)
Supplement: Figure S8 — Mediation analyses [file bcd-24-0355_figure_s8_suppsf8.pdf]

Figure S8. Mediation analyses

(A) Acetaldehyde for myeloid neoplasms mortality

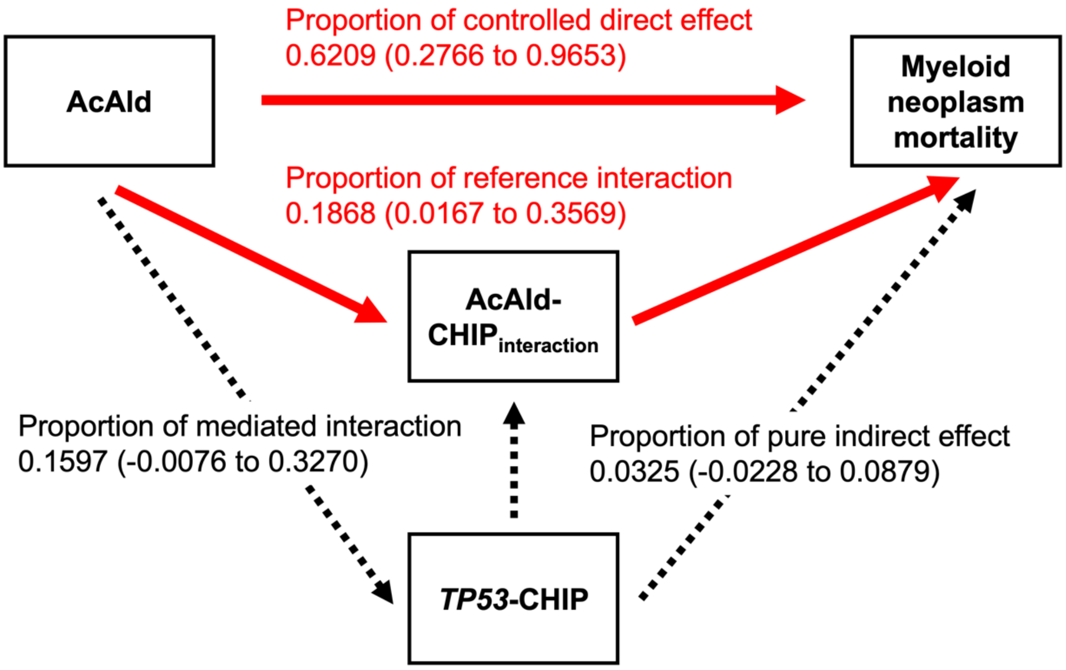

(B) Smoking for respiratory disease mortality

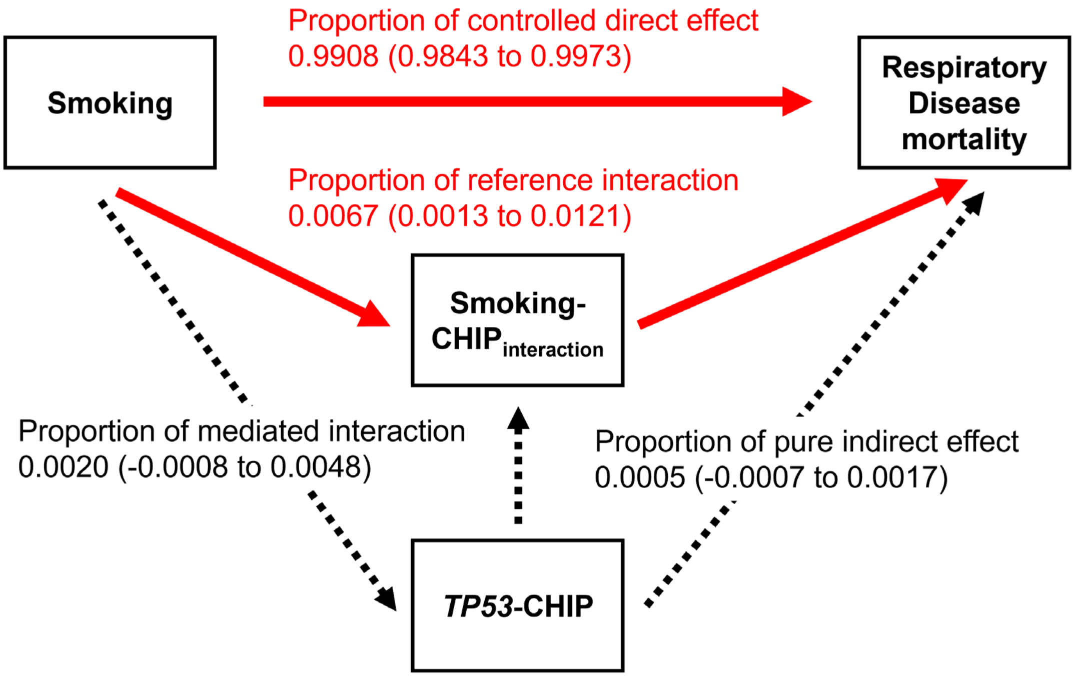

To describe the relationship among environmental factors, *TP53*-CHIP, and disease-specific mortality, we performed mediation analyses by using Stata command *med4way*(1). We applied a Cox regression model for disease-specific mortality and a logistic regression model for *TP53*-CHIP, respectively. Proportions yield

significance (P < 0.05) are shown in red. (A) Both models were adjusted for age, sex, drinking habits, alcohol consumption, smoking habits, Brinkman index, body mass index, comorbidities (hyperlipidemia, hypertension, diabetes, and cancer), and *ALDH2*. Individuals prone to acetaldehyde exposure were defined as individuals with both rs671 Lys+ and ever-drinker status. (B) Both models were adjusted for age, sex, drinking habits, alcohol consumption, Brinkman index, body mass index, and comorbidities (hyperlipidemia, hypertension, diabetes, and cancer). Smoking status was defined as ever-smoker.

Mediation analyses show how much of the overall effect of environmental factors on disease-specific mortality is attributable to *TP53*-CHIP when the total impact of environmental factors is set at 1. When the proportion of controlled direct effect shows 1, *TP53*-CHIP does not mediate the effects of environmental factors on disease-specific mortality, which indicates that *TP53*-CHIP has an independent impact on disease-specific mortality, separate from the impact of each environmental factor. Mediated interaction indicates an additive interaction that operates only if the environmental factor has an effect on *TP53*-CHIP. Reference interaction indicates an additive interaction that operates only if *TP53*-CHIP is present.

Reference

1. Discacciati A, Bellavia A, Lee JJ, Mazumdar M, Valeri L. Med4way: a Stata command to investigate mediating and interactive mechanisms using the four-way effect decomposition. Int Int J Epidemiol **2019**;48(1):15-20 doi 10.1093/ije/dyy236.
