# Supplementary material for: Clinical Significance of TP53-Mutant Clonal Hematopoiesis Across Diseases
Source: Blood Cancer Discov. 2025 Jun 17;6(4):298–306. doi: 10.1158/2643-3230.BCD-24-0355 (PMC12209765; doi:10.1158/2643-3230.BCD-24-0355)
Supplement: Figure S9 — Clinical impact of TP53-CHIP by VAF [file bcd-24-0355_figure_s9_suppsf9.pdf]

Figure S9. Clinical impact of *TP53*-CHIP by VAF

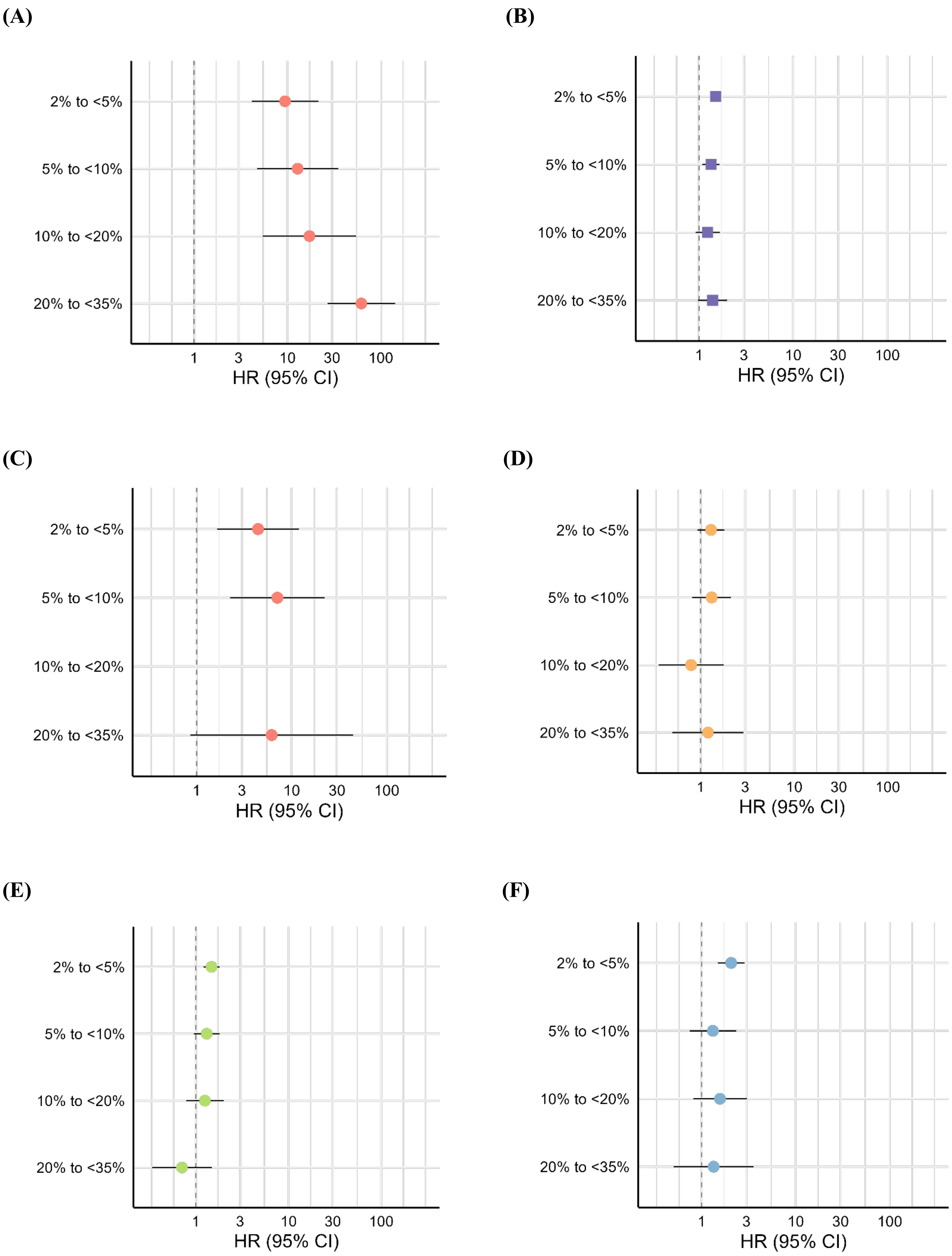

TP53-CHIP risk of mortality (A) myeloid neoplasms (number of events: 162); (B) overall (number of events: 29,626); (C) lymphoid neoplasms (number of events: 199); (D) cardiovascular disease (number of events: 5,645); (E) non-hematological neoplasms (number of events:13,256); (F) non-neoplastic respiratory disease (3,405) are shown.

Hazard ratios (HRs) and their 95% confidence intervals (CIs) were estimated using Cox proportional hazard models of disease-specific mortality adjusted for age, sex, drinking habits, alcohol consumption, smoking habits, Brinkman index, body mass index, and comorbidities (hyperlipidemia, hypertension, diabetes, and cancer).
