# Supplementary material for: Clinical Significance of TP53-Mutant Clonal Hematopoiesis Across Diseases
Source: Blood Cancer Discov. 2025 Jun 17;6(4):298–306. doi: 10.1158/2643-3230.BCD-24-0355 (PMC12209765; doi:10.1158/2643-3230.BCD-24-0355)
Supplement: Figure S10 — Probability of overall survival by VAF [file bcd-24-0355_figure_s10_suppsf10.pdf]

Figure S10. Probability of overall survival by VAF

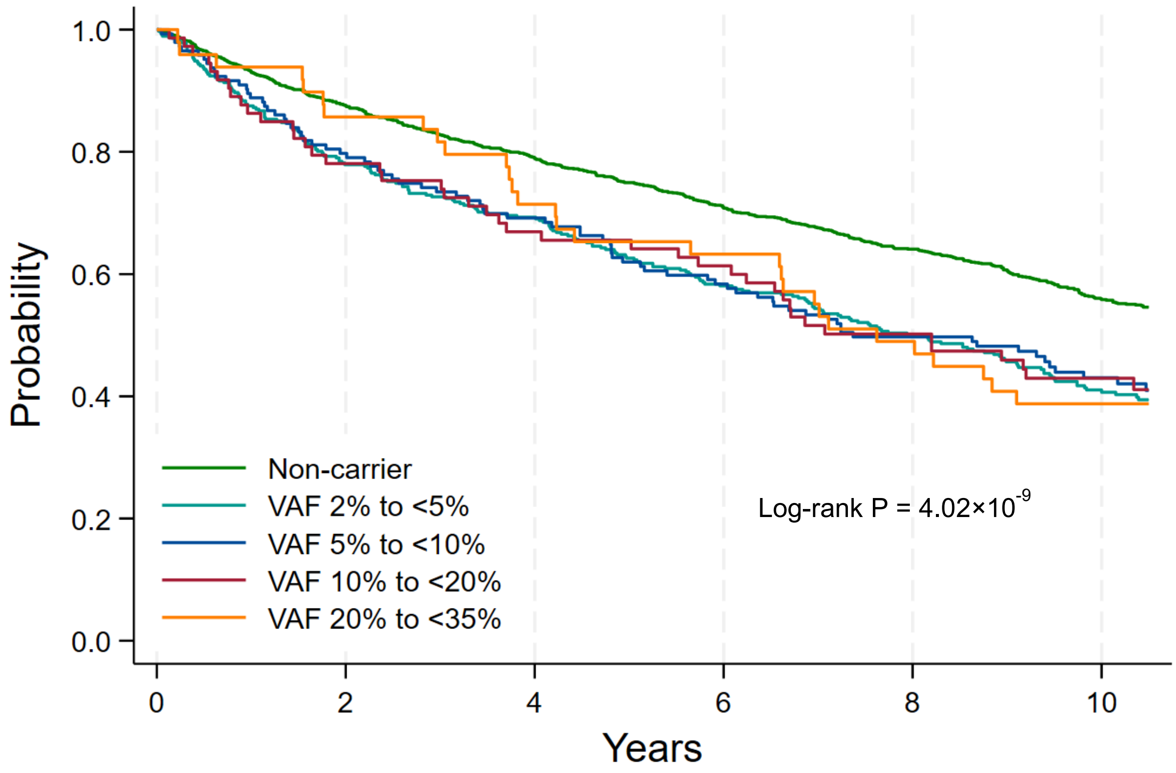

Number at risk						
Non-carrier	1276	1096	984	881	789	524
VAF 2% to <5%	372	282	249	204	174	110
VAF 5% to <10%	144	114	96	81	67	46
VAF 10% to <20%	73	57	48	44	36	26
VAF 20% to <35%	49	42	35	31	24	17

The probability of overall survival was estimated using the Kaplan–Meier method and compared using the log-rank test. Non-carriers were selected using 1:2 exact matching based on age and sex (mean age [standard deviation]: 72.50 [8.83]; proportion of males: 71.63% in both carriers and non-carriers).
