# Supplementary material for: Clinical Significance of TP53-Mutant Clonal Hematopoiesis Across Diseases
Source: Blood Cancer Discov. 2025 Jun 17;6(4):298–306. doi: 10.1158/2643-3230.BCD-24-0355 (PMC12209765; doi:10.1158/2643-3230.BCD-24-0355)
Supplement: Figure S11 — Performance of mutation call [file bcd-24-0355_figure_s11_suppsf11.pdf]

Figure S11. Performance of mutation call

(A)

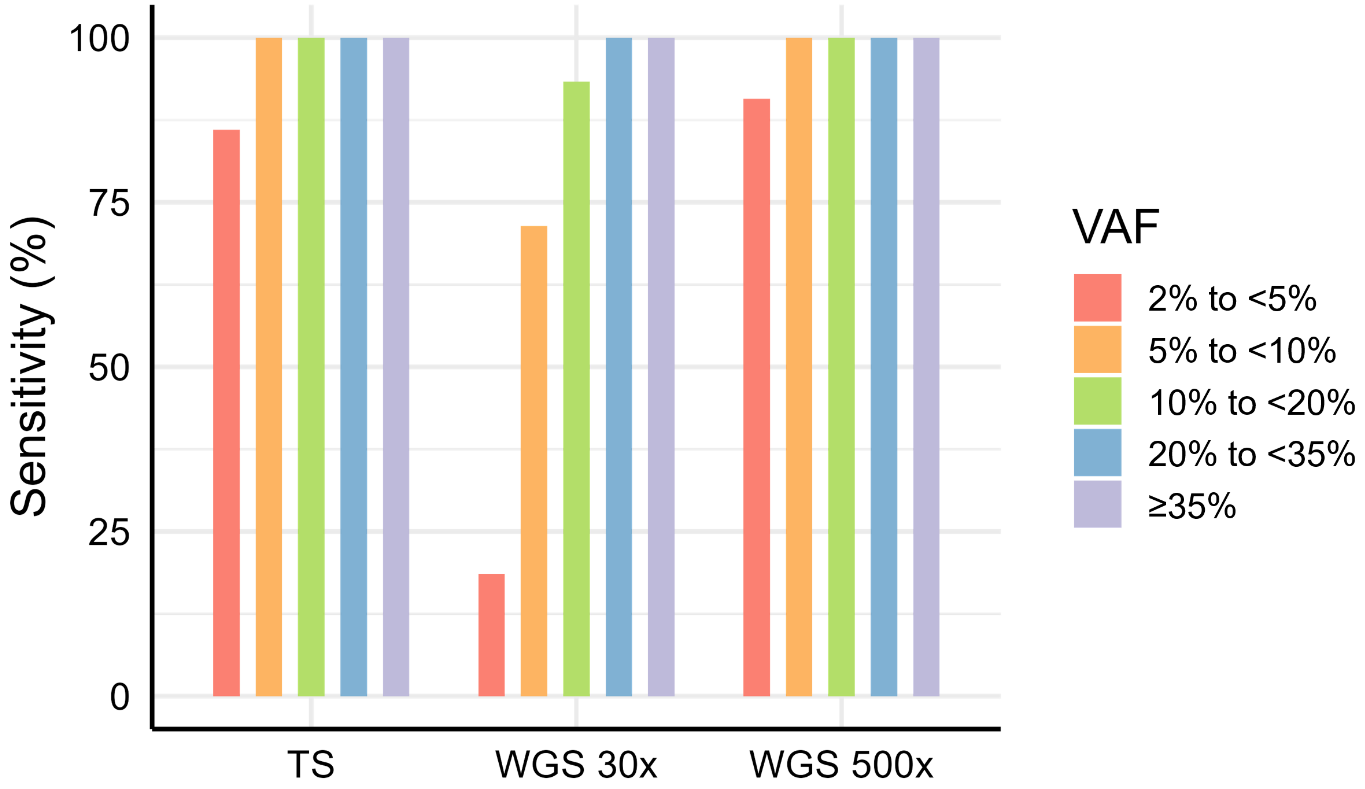

(B)

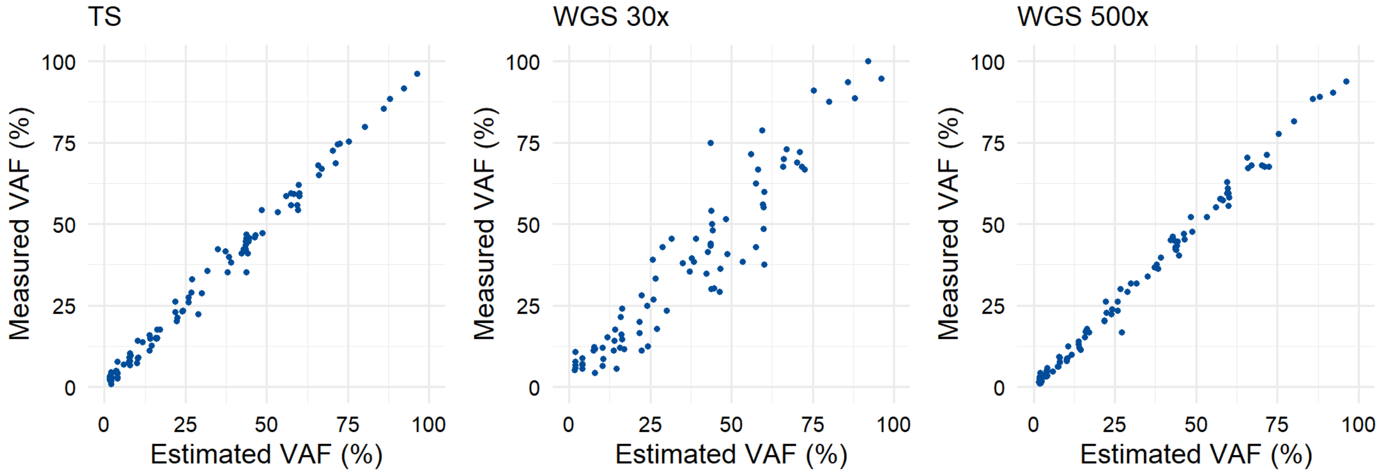

Targeted sequencing (TS) and high-coverage (500x) whole-genome sequencing (WGS) were performed for the sample created by mixing multiple samples to check call performance.

(A) Comparable to high-coverage WGS in terms of sensitivity. (B) Comparable to high-coverage WGS in terms of accuracy of detected mutations.
